# ROS-responsive cellular vesicles with ferroptosis-targeting siACMSD delivery for acute kidney injury therapy

**DOI:** 10.7150/thno.119667

**Published:** 2026-01-01

**Authors:** Yunjing Zhang, Qing Deng, Yangtao Xu, Wei Wu, Tian Wu, Jia Huang, Yugang Hu, Weiqiang Lin, Ximing Xu, Jicheng Wu

**Affiliations:** 1 Department of Ultrasound, Renmin Hospital of Wuhan University, Wuhan 430060, China.; 2 Cancer Center, Renmin Hospital of Wuhan University, Wuhan 430060, China.; 3 Department of Intensive Care Unit, Renmin Hospital of Wuhan University, Wuhan 430060, China.; 4 Department of Nephrology, Center for Regeneration and Aging Medicine, The Fourth Affiliated Hospital of School of Medicine and International School of Medicine, International Institutes of Medicine, Zhejiang University, Yiwu 322000, China.

**Keywords:** reactive oxygen species response release, cellular vesicles, acute kidney injury, ferroptosis, ACMSD

## Abstract

**Background:** Acute kidney injury (AKI) is a severe and prevalent nephrotic syndrome which lack of definitive therapies. Alpha-amino-β-carboxymuconic acid-ε-semialdehyde decarboxylase (ACMSD) is a metabolic enzyme mainly expressed in the kidney which exacerbated AKI injury by promoting TCA cycle and inhibiting nicotinamide adenine dinucleotide (NAD^+^) production, whereas lack of effective intervention strategies for ACMSD-targeted therapy.

**Methods:** Herein, we knocked out ACMSD *in vitro* through CRISPR-Cas9 method, and developed a reactive oxygen species (ROS)-responsive neutrophil-derived cellular vesicles (CVs) drugs (RNAi@ROS-CVs), which efficiently mediated ACMSD knockdown *in vivo*, exploring the mechanism of ACMSD-induced ferroptosis process in AKI.

**Results:** ACMSD knockout effectively alleviated cisplatin (CP)-induced mitochondrial damage, suppressed TCA cycle progression, promoted NAD^+^ synthesis, and inhibited ferroptosis in HK2 cells. In mice AKI model, RNAi@ROS-CVs effectively targeted the injured kidneys, downregulated ACMSD expression in renal tubular epithelial cells, reduced ROS production and lipid peroxidation, and alleviated CP or ischemia/reperfusion (I/R)-induced ferroptosis.

**Conclusion:** These findings highlight the therapeutic potential of ACMSD-targeted knockout in AKI intervention and introduce a versatile and efficient controlled-release drug delivery platform for AKI-targeted therapy, with potential applicability to other acute renal diseases.

## Introduction

Acute kidney injury (AKI) is a prevalent clinical syndrome characterized by a sudden loss of renal function, and has increasingly become a serious global health problem due to its high morbidity and mortality rates 1. Numerous factors such as nephrotoxic drugs, ischemia/reperfusion (I/R) injury, acute upper urinary tract obstruction, and sepsis have been demonstrated to trigger or aggravate the AKI 2. Nevertheless, it is unclear which regulators initiate and exacerbate AKI and what types of drugs are effective in therapy 3, 4. Furthermore, recent studies have confirmed that patients who do not completely recover from AKI are subsequently prone to develop underlying chronic kidney disease (CKD) and eventually irreversible end-stage renal disease, which is associated with significant short-term and long-term mortality 5. Therefore, there exists an urgent need to gain a deep understanding of the complex regulatory mechanisms that drive the occurrence and development of AKI and set the molecular theoretical foundation for therapeutic intervention at the molecular level.

Previous research has shown that ferroptosis is involved in the process of injury of renal tubular cells and in the reduction of renal blood flow, representing an important driver and regulator of AKI development 6. Ferroptosis is a form of cell death caused by iron-dependent generation of reactive oxygen species (ROS) and lipid peroxidation driven by Fe^2+^ redox cycling 7, 8. These two processes impair the cell mitochondrial function in the cell, which blocks the aerobic oxidation pathway and ATP supply regulated by mitochondria, hindering the repair of damaged cells and further aggravating the degree of ferroptosis-mediated AKI. Nicotinamide adenine dinucleotide (NAD^+^) is a cosubstrate for various enzymes in the mitochondrial metabolism, including the sirtuin family of NAD^+^-dependent protein deacylase. The augmentation of NAD^+^ levels and the activation of sirtuin are vital for the maintenance of mitochondrial homeostasis and clearance of lipid metabolites, as well as for the organismal metabolism and lifespan of organisms 9, 10. A reduction in NAD^+^ levels causes sirtuin deactivation, lipid oxidation intensification, and mitochondrial homeostasis disruption, which aggravates the injury-related diseases such as AKI progression and the injury deterioration 11. Alpha-amino-β-carboxymuconic acid-ε-semialdehyde decarboxylase (ACMSD), a metabolic regulatory enzyme primarily expressed in the liver and kidneys, limits the proportion of ACMS capable of undergoing spontaneous cyclization in the de novo NAD^+^ synthesis pathway 12-14. Studies have confirmed that, an increase in ACMSD expression alters the balance of ACMS from the NAD^+^ biosynthesis to acetyl-CoA production, activating the mitochondrial TCA cycle and generating ROS, ultimately causing the incidence and progression of mitochondrial-related diseases such as AKI 15. However, a reduction in ACMSD expression stimulates NAD^+^ synthesis and SIRT1 activity, protecting mitochondria from damage and lipid peroxidation 16. Our previous study suggested that ACMSD expression in renal tubular epithelial cells considerably increased in ferroptosis-induced injury. The upregulation of ACMSD in AKI suggests that the inhibition of this enzyme has a potential therapeutic application 17. In fact, a series of potent ACMSD inhibitors with high therapeutic interest in terms of protecting kidneys from injury have been explored 12. Nevertherless, for potential future applications in clinical settings, it is essential to clatify how to effectively and precisely inhibit ACMSD expression at the site of kidney injury.

A particularly promising avenue involves the use of extracellular vesicles (EVs), natural nanoparticles released by cells that inherit surface-associated biomolecules and internal loading drugs for precise delivery and targeted therapy 18, 19. EVs possess high biocompatibility, targeting efficiency, penetration ability, prolonged systemic circulation, and abundant drug-loading capacity 20. Through a process of sanitation and extrusion, a substantial quantity of EVs in the form of nanoscale cellular vesicles (CVs) could be generated efficiently 21. These CVs provide a versatile platform for the modification and incorporation of functional compounds 22. Moreover, CVs derived from different cells possess specific biological characteristics, rendering them particularly attractive for applications in disease diagnostics and combination therapies 23. Neutrophils are the most abundant cell type in the blood circulation and the first cells recruited to the site of inflammation 24. AKI is accompanied by a significant inflammatory response, especially in the early stage, when neutrophils instantly migrate to the injured renal tubules 25. Therefore, neutrophil-derived CVs (Neu-CVs) are naturally effective in targeting inflammatory diseases. Nonetherless, an efficient and rapid drug release is also crucial for the efficacy of AKI-targeted therapy because AKI progresses rapidly and tissue damage often becomes irreversible within a short period of time 26. Precise targeting effects combined with an efficient drug-release system help in rapidly blocking disease progression 27, 28. ROS accumulation is the most prominent feature of ferroptosis and AKI in the renal tissue. An ROS-responsive drug delivery system can rapidly block AKI progression by increasing the drug concentration in the lesion area through safe and controllable transient drug release 29.

**Figure 1:**
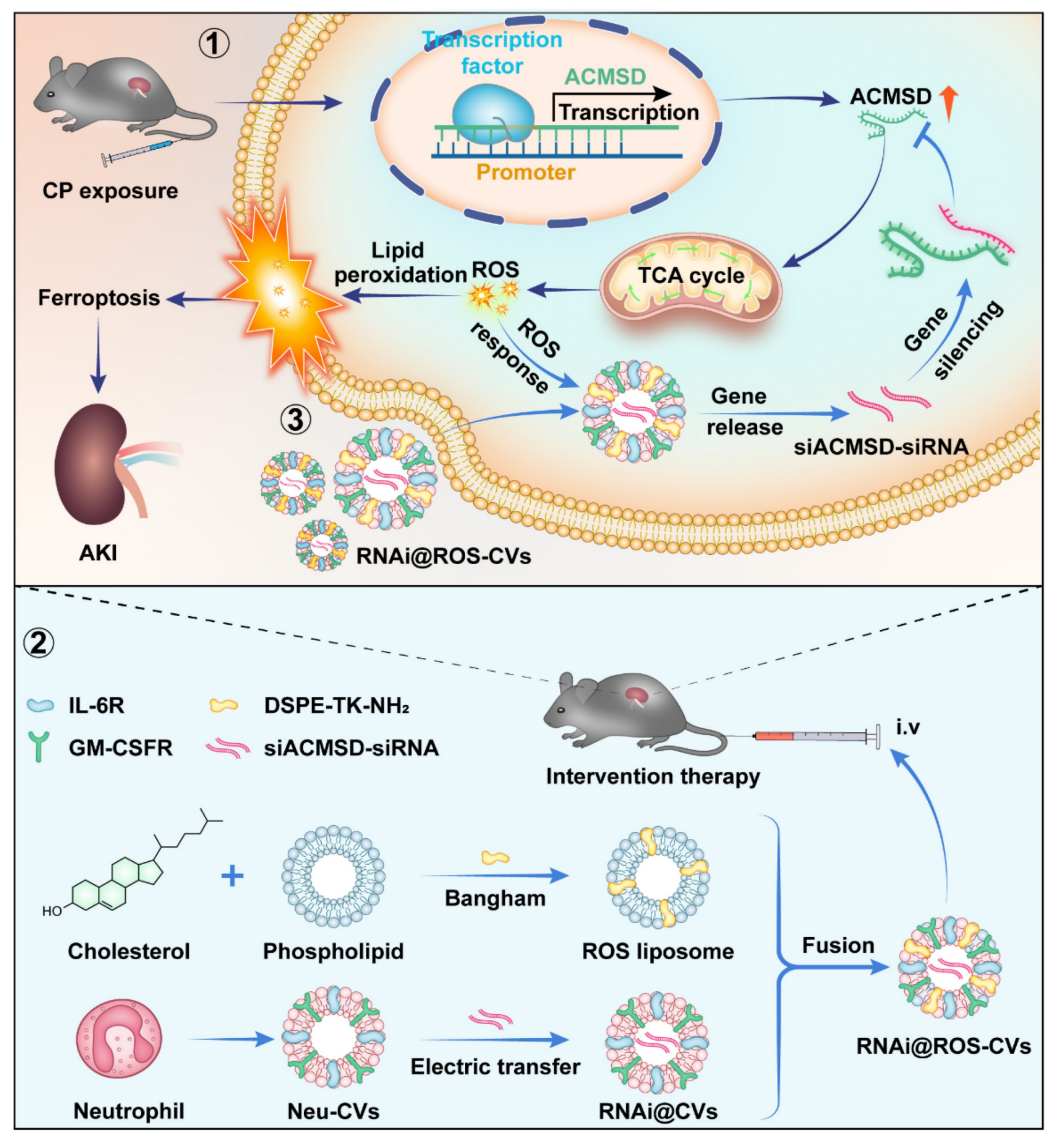
Molecular mechanism underlying ACMSD-regulated ferroptosis for AKI and targeted intervention strategy against accelerating AKI. ACMSD accelerates the process of ferroptosis and promotes the AKI by stimulating TCA cycle, increasing ROS accumulation and lipid peroxidation. Our engineered neutrophil-derived CVs act against ferroptosis and AKI therapy *via* the targeted blockade of the ACMSD-TCA axis.

Herein, we found that ACMSD knockout considerably alleviated CP-induced mitochondrial damage, inhibited TCA cycle progression, promoted NAD^+^ synthesis, and inhibited ferroptosis in HK2 cells. Moreover, we successfully constructed the ROS-responsive neutrophil cellular vesicles (ROS-CVs) by adopting the lipid membrane fusion strategy. The siRNA targeted for ACMSD delivered *via* ROS-responsive neutrophil-derived CVs (RNAi@ROS-CVs) were shown to effectively mediates the knockdown of ACMSD in HK2 cells *in vitro* and alleviates the ACMSD-regulated ferroptosis process. In mice AKI model, RNAi@ROS-CVs effectively targeted the injured kidneys, knocked down the ACMSD expression in renal tubular epithelial cells, reduced ROS production and lipid peroxidation, and alleviated cisplatin- or ischemia/reperfusion (I/R)-induced ferroptosis (Figure 1). Our study revealed the positive role of ACMSD-targeted knockout in the therapeutic intervention for AKI, thereby providing a universal and efficient controlled-release drug delivery system that is also suitable for the treatment of various other acute kidney diseases.

## Materials and Methods

### Materials

DMEM medium (Gibco, C11995500BT), RPMI1640 medium (Gibco, C11875500BT), fetal bovine serum (Gibco, 10099-141C), penicillin-streptomycin (Gibco, 15140122), cell proliferation dye eFluor™ 670 (Invitrogen, 65-0840-85), eBioscience™ CFSE (Invitrogen, 65-0850-84), Alexa Fluor™ 647 anti-rabbit IgG (Invitrogen, A21244), C11 BODIPY^TM581/591^ (D3861) were purchased from Thermo Fisher. The primary antibody 4HNE (ab48506), ACMSD (ab48506), IL-6R (ab271042), IL-1R (ab317740), CXCR4 (ab181020), CCR2 (ab273050) and Na^+^K^+^-ATPase (3010S) were purchased from Abcam or Cell Signaling Technology. PTGS2 (ET1610-23) and GPX4 (ET1706-45) were purchased from Huabio Biological Technology. CPI613 (Cat# 95809-78-2) were obtained from Sigma. DOTAP, DOPE, cholesterol, 2,7-Dichlorodi-hydrofluorescein diacetate (DCFH-DA) were purchased from Sigma. DSPE-TK-PEG_2000_ was procured from Qiyue Biological Company (Xi'an, China).

### ROS-responsive liposomes (Lips)

The ROS-responsive liposomes were synthesized by the thin-film hydration technique. Briefly, a mixture containing 5 mg DOTAP, 5.3 mg DOPE (DOTAP: DOPE = 1:1 molar ratio), 5 mg DSPE-TK-PEG_2000_, and 2.77 mg cholesterol was dissolved in 8 mL trichloromethane in a round-bottom flask. Then, the mixture was subjected to rotary evaporation at 110 rpm and 0.06-0.08 MPa vacuum in a 40 °C water bath to remove the organic solvent. The residual solvent traces were further eliminated by subjecting the solution to a vacuum pressure of 0.1 MPa for one hour at 100 rpm. The resulting dried lipid film was then hydrated with 10 ml PBS at 40 °C for 20 minutes to form a homogeneous emulsion. Finally, the liposomes were obtained by extruding the mixture through a polycarbonate membrane (Whatman, Life Science) with a pore size of 0.4 μm to reduce particle size.

### Preparation of neutrophil membranes

The mouse neutrophils were extracted from peripheral blood of C57BL/6 mice with peritoneal inflammation induced by LPS (1.5 mg/kg, stimulated for 6 h) and purified by peripheral blood neutrophils isolation kit. Neutrophil count, purity, and morphological differentiation were assessed using blood counts, Wright-Giemsa staining, and flow cytometry. The purified neutrophils were then subjected to lysis using a precooled hypotonic buffer. The hemocytometry, wright-giemsa staining and flow cytometry were used to assess neutrophil counts, purity, and granulocytic morphologic differentiation.

### Preparation and characterization of Neu-CVs

The preparation and characterization of Neu-CVs according to a protocol reported previously 30. Briefly, the neutrophil cells were initially suspended in a ddH_2_O and disrupted with liquid nitrogen repeatedly. Then the mixture was added 150 ng DNase and RNase (Invitrogen) and centrifuged at 3500 g for 15 min at 4 °C. Then we collecting the resulting supernatants again and further centrifuged it at 20,000 g for 30 min in 4 °C condition. Finally, the supernatant was centrifuged at 100,000 g for 2 hours to collect the cellular membranes. Washing the sediment three times with PBS containing protease inhibitor tablets and centrifugation, resuspend the sediment in cold PBS. Using a mini-extruder, the cellular membranes was successively passed through polycarbonate porous membranes of 800 nm, 400 nm, and 200 nm. The particle size and potential of extracellular vesicles were evaluated using dynamic light scattering (Zetasizer Pro) and nanoparticle tracking analysis (NTA, NanoSight Pro). Nanovesicle morphology was investigated using negative staining TEM (JEM-2010HT, JEOL, Japan). Protein concentrations of CVs were determined using a Bicinchoninic Acid Kit (Sigma-Aldrich).

### Preparation of siRNA loaded Neu-CVs

The siRNA for ACMSD were loaded into Neu-CVs by electroporation. Briefly, the neutrophil CVs were resuspended in electroporation buffer, which contains 1.15 mM potassium phosphate (pH 7.2), 25 mM potassium chloride, and 21% OptiPrep working solution. Then the cellular membranes were extruded through 200-nm polycarbonate to Neu-CVs. Subsequently, the siRNA was added to Neu-CVs at a 4:1 ratio (4 μmol siRNA in 1 mg protein concentration of CV, 1 mg/mL) and then electroporated using Gene Pulser Xcell (Bio-Rad) to form exosomal siRNA complexes. After electroporation, the CVs were centrifuged at 4 °C at 10000 rpm for 2 hours to remove the excessive siRNA, and the precipitation was then suspended in a cold PBS solution. The remaining content of siRNA in supernatant was determined by DNA gel-electrophoresis and the High Performance Liquid Chromatography (HPLC).

### Preparation and characterization of ROS responsive CVs

To obtain the ROS-responsive RNAi@ROS-CVs NPs, we mixed the ROS-Lip with RNAi@Neu-CVs at a 2:1 mass ratio. The ROS responsive CVs drugs were obtained by repeated extrusion and fusion of ROS-responsive lipids into RNAi@ROS-CVs. The particle size and potential of extracellular vesicles were measured using dynamic light scattering (Zetasizer Pro) and Nanovesicle morphology was investigated using negative staining TEM (JEM-2010HT, JEOL, Japan).

### HPLC analyses for siRNA

To detect the siRNA in the supernatant post-reaction, the Agilent 1100 system (Santa Clara, CA) with a C18 column (5 μm, 250 × 4.6 mm, Torrance, CA) was utilized. A 20 min isocratic elution under 30 °C was recommended, and the flow rate was recommended to be 0.5 mL/min. In addition, the recommended separation solvent was 0.1% TFA in water (solvent A): 0.1% TFA in acetonitrile (solvent B) (1:1 v/v). The chromatograms were obtained at 280 and 214 nm.

### ACMSD silencing assays

The siRNA sequences targeting ACMSD were as follows: sense: 5'-GGAGCUCUU-UCCUGUCUAUTT-3', antisense: 5'-AUAGACAGGAAAGAGCUCCTT-3'. For *in vitro* transfection, the CVs were electroporated with siRNA as described above. Next, 2×10^5^ HK2 cells were prepared in a 6-well plate and treated with RNAi@ROS-CVs, and subsequently washed with PBS before further analysis.

### Cell culture

The human kidney cell line HK2 was obtained from ATCC and cultured at 37 °C in a humidified atmosphere of 5% CO_2_. The cells were maintained in standard RPMI 1640 medium (Gibco) supplemented with 10% FBS (Gibco) and 100 U/ml penicillin/streptomycin. All the cells were tested negative for mycoplasma contamination using a mycoplasma detection kit before used.

### ACMSD knockout by CRISPR/Cas9 assay

The guide RNAs targeted for ACMSD were designed and integrated in the LentiCRISPRv2 KO plasmid. The transfection for ACMSD knockout were performed as reported previously 31. Briefly, a mixture of single sgRNA vectors, envelope vector pMD2.G, and packaging vector psPAX2 at a 4:3:1 ratio was prepared in OPTI-MEM (Thermo Fisher Scientific) with PEI (Polysciences), and transfected into HEK293T cells instantaneously. The HK2 cells which was in the logarithmic growth phase were then infected overnight with the lentivirus expressing Cas9 and sgRNAs targeting ACMSD, in the presence of 5 µg/mL polybrene. The medium was replaced after 8 h and new medium containing puromycin selection (250 ng/mL) was added and cultured for another three days.

### Cell viability

For CCK-8 assay, 3 × 10^3^ HK2 cells were inoculated into a 96-well plate and added cisplatin (CP, 20 μM) to continue incubate for 24 h. Subsequently, 10 μL of CCK-8 reagent (Dojindo, Cat# CK04) was added to each well and absorbance was measured at a wavelength of 450 nm.

### Live/dead cell staining

The live and dead cells were detected using the LIVE/DEAD Kit (Thermo Fisher Scientific, Cat# 40747ES76). Briefly, the HK2 cells with appropriate density were exposed to CP with or without RNAi@ROS-CVs (50 μg/mL) for 24 h. Next, the Calcein-AM and PI (10 μM) were used to label the cell viability and the images of cell morphology were captured by confocal microscope (TCS SP8, Germany).

### Animal model

All animal experiments were performed and approved according to the guidelines of the Animal Care and Use Committee of Renmin Hospital of Wuhan University (20241201A). The AKI model was induced in male C57BL/6J mice aged 6-8 weeks through cisplatin and ischemia-reperfusion injury (IRI). The CP-induced AKI model involved intraperitoneal injection of cisplatin (20 mg/kg) once, the folic acid nephropathy was induced by a single *i.p.* injection of FA (200 mg/kg) in 0.3 mol/L sodium bicarbonate 32. The RNAi@ROS-CVs (7.5 mg/kg) or PBS were administered via the tail vein 30 min before CP injection and every 24 h for consecutive three days. For IRI-induced AKI model, the method was performed as previously 17. The dose of therapeutic agents such as RNAi@ROS-CVs and PBS were consistent in AKI model induced by CP, and blood samples were collected. Drug therapeutic effect was evaluated through the serum creatinine and pathologic evaluation.

### Cell mitochondria stress test

The metabolic profile of cells was performed using a Seahorse XF-96 extracellular flux analyzer (Agilent Seahorse Bioscience, USA), as described in a previous study 33. Briefly, 1.0 × 10^4^ HK2 cells or HK2^ACMSD-KO^ cells were seeded in 96-well plates (Agilent, 102601-100) and treatment with fresh RMPI 1640 medium (Agilent, 103681-100; pH 7.4) containing with PBS, CP (20 μM) or CP + RNAi@ROS-CVs (50 μg/mL) for 24 h. After that, 2.5 μM oligomycin, 2 μM FCCP, and 0.5 μM rotenone and antimycin A were injected in order. The oxygen consumption (OCR) values were measured in 6 wells per sample.

### Assessment of renal injury

For evaluation, the paraffin-embedded sections (4 µm) were stained with either hematoxylin and eosin (H&E) or periodic acid-Schiff (PAS) as described in the instructions. Pathological damage in the mouse was assessed by different expert pathologists using light microscopy in a blinded manner, grading based on parameters such as proximal tubule dilation, brush-border damage, proteinaceous casts, interstitial widening, and necrosis (0, none; 1, <11%; 2, 11-25%; 3, 26-45%; 4, 46-75%; 5, >75%).

### Immunohistochemistry (IHC)

The IHC analysis was conducted following the methodology described in a previous study 33. Briefly, tissue sections (4 µm) were deparaffinized and subjected to antigen retrieval, followed by quenching of endogenous peroxidase activity prior to incubation with primary antibodies. After incubation and elution, the HRP-labeled secondary antibodies (Abcam) were used for detection, and the sections were counterstained with hematoxylin.

### Immunofluorescence

After fixation with paraformaldehyde, the HK2 cells and kidney sections were incubated with primary antibodies against ferritin 1 (FTH-1), 4-hydroxynonenal (4-HNE), and prostaglandinendoperoxide synthase 2 (PTGS2). Subsequently, the cells were subjected to secondary antibody. After treated with DAPI, the immunofluorescence images were obtained by confocal laser microscopy (FV3000, Olympus).

### Tissue iron assay

The tissue iron content was determined by iron assay kit (A039-2-1, Nanjing, China) following the instructions. Briefly, the kidney tissues were weighted and add to same volume saline for homogenate. Subsequently, the samples of supernatant were processed and added with specific reagents according to the kit steps, then boiled, cooled, and centrifuged at 3500 rpm for 10 min to obtain the new supernatant. The tissue iron content was quantified by measuring absorbance at 520 nm using a microplate reader.

### Transmission electron microscopy analysis

The renal tissues or HK2 cells were fixed in 2.5% glutaraldehyde overnight, followed by post-fixation in 1% osmium tetroxide fixation, gradient dehydration, infiltration, embedding, sectioning, and finally observation with a Hitachi H-750 Bio-TEM.

### Preparation of DiR-labeled CVs

The DiR dye (Thermo Fisher) was dissolved in DMSO at a concentration of 10 μM. Then, the same number of particles (measured by NTA) of ROS-Lip, ROS@Neu-CVs, or RNAi@ROS-CVs were resuspended in 1 mL PBS, followed by the addition of 0.1 μM DiR dye. Then, the mixture was shaken and incubated at 37 °C for 20 min, after which it was ultrasonicated in a water bath for 5 min. Finally, the DiR labeled ROS-Lip, ROS@Neu-CVs or RNAi@ROS-CVs were used for pharmacokinetics and biodistribution studies *in vivo*.

### Drug metabolism assessed by IVIS imaging

The AKI model of C57 mice were established by intraperitoneal injection of CP (20 mg/kg), each group of mice were *i.v.* injected with 150 μL PBS containing ROS-Lip, ROS@Neu-CVs, or RNAi@ROS-CVs respectively. At different time after drug exposure, the fluorescence signals on AKI kidney or main organs were assessed with an IVIS imaging system (IVIS, Xenogen Corp, Alameda, CA) after the mice were euthanized and dissected.

### The location of RNAi@ROS-CVs in the kidneys

The AKI mice or healthy control were *i.v.* administered with DiR-labeled ROS-Lip, ROS@Neu-CVs, or RNAi@ROS-CVs. At 24 h after drug exposure, the mice were euthanized and the kidneys were embedded with OCT (LEICA) and subjected to frozen sections (8 μm). After fixation with paraformaldehyde, the sections were stained with lotus tetragonolobus lectin (LTL) (FL#1321, Vector) and DAPI in the dark. The sections were photographed under a confocal laser microscope (Wetzlar, Germany).

### Safety evaluation for CV drugs

To test the biosafety of CV drugs *in vivo*, we conducted the RNAi@ROS-CVs intravenous injection for C57 mice with a dose of 10 mg/kg for consecutive seven days. The individual index which related to the main organ function were detected by biochemistry analyzer, when the mice were euthanized and the peripheral blood or main organs were collected.

### qRT-PCR Assay

The trizol reagent (AG, Hunan, China) was used to extract total RNA, which was then reverse transcribed into cDNA using the PrimeScript RT kit (Vazyme Biotech, Cat# R423) according to the manufacturer's instructions. The results were calculated using the 2^-ΔΔt^ method.

### Western blot

The protein samples with loading buffer were denatured and isolated on SDS polyacrylamide gel. Next, the segregated proteins were transferred onto a nitrocellulose membrane using a Power Blotter (Invitrogen), incubated with primary antibodies and HRP-conjugated secondary antibody respectively.

### The JC-1 staining assay

The HK2 cells were pre-treated with CP or CP with/without RNAi@ROS-CVs for 24 h, followed by the addition of 2 μM JC-1. After dark incubation, mitochondrial membrane potential depolarization was assessed using a flow cytometer or confocal microscope (TCS SP8, Germany) on excitation wavelength of 585/514 nm.

### The GSH levels assay

After different treatments, the HK2 cells were collected and resuspended in special buffer. The GSSG assay kit (Cat# A006-2) were used to detect the GSH concentration in cell lysis. Simultaneously, the monobromobimane (MBB) probe (MCE, Cat# HY-100041, 15 μM) were used to detect the GSH level in HK2 cells. The MBB probe fluorescence intensity was detected by confocal laser scanning microscope.

### Intracellular ROS level assay

The MitoSOX (Invitrogen, Cat# M36008, 10 μM) and C11 BODIPY^TM581/591^ (Thermo Fisher, Cat# D3861, 10 μM) or dihydroethidium (DHE) probe (MCE, Cat# HY-D0079, 10 μM) were used to detect the intracellular ROS levels in HK2 cells. The flow cytometer and confocal microscope were used to evaluate the positive staining of HK2 cells after different treatment.

### Cellular iron level analysis

The free iron levels in HK2 cells were determined using the labile iron pool (LIP) assay (MCE, Cat# HY-D0041) and the Phen Green™ SK (PGSK) probe (Thermo Fisher, Cat# P14313) according to the methods described. The flow cytometric analysis (BD Biosciences) and confocal microscope (Wetzlar, Germany) were performed to acquire in situ intracellular iron levels and fluorescence intensity in HK2 cells.

### ELISA assay

The standard level of IL-1β, IL-13 in supernatant after Neu-CVs pretreatment was quantified using ELISA kits (KE10003, KE10021). The quantitative detection of inflammatory factors in the AKI kidney was carried out according to the instructions of the ELISA kit. In short, a certain mass of fresh kidney tissue was divided, weighed and added a quantitative lysis solution for crushing. After centrifugation at 8,000 g for 30 min at 4 °C, the supernatants were collected for the detection of IL-6, IL-1, CXCL-2, CXCL-8 and CXCL-12 using ELISA kits (Servicebo).

### Mitochondrial morphology

The MitoTracker Green probe (Beyotime, Cat# C1049; 200 nM) were used to observe he Mitochondrial morphology and mitochondrial targeting effect. Briefly, the HK2 cells were incubated with MitoTracker DeepRed staining in CP with or without RNAi@CVs groups. The images were captured by a confocal laser scanning microscope.

### scRNA-seq data analysis

The expression of ACMSD in AKI model which induced by unilateral renal ischemia/reperfusion (IR) were acquired from the single cell database (https://www.ncbi.nlm.nih.gov/geo/query/acc.cgi?acc=GSE139506/GSE220675), and UMAP analysis was also performed on the website as described previously 34. The cell-type clusters and marker genes were identified using the R version 3.6.1 library Seurat version 3.1.0.

### Statistical analysis

The GraphPad Prism software (version 8.5; www.graphpad.com) was used for all statistical analyses. The comparisons between two groups were conducted using an unpaired two-tailed t-test, the multiple group comparisons were analyzed by ordinary one-way or two-way ANOVA followed by either Tukey's or Dunnett's post hoc test. *P* values ≤ 0.05 were considered as statistically significant differences; “ns” represents no significance; *P < 0.05, **P < 0.01 and ***P < 0.001.

## Results

### Knockout of ACMSD alleviate CP-induced ferroptosis *in vitro*

**Figure 2:**
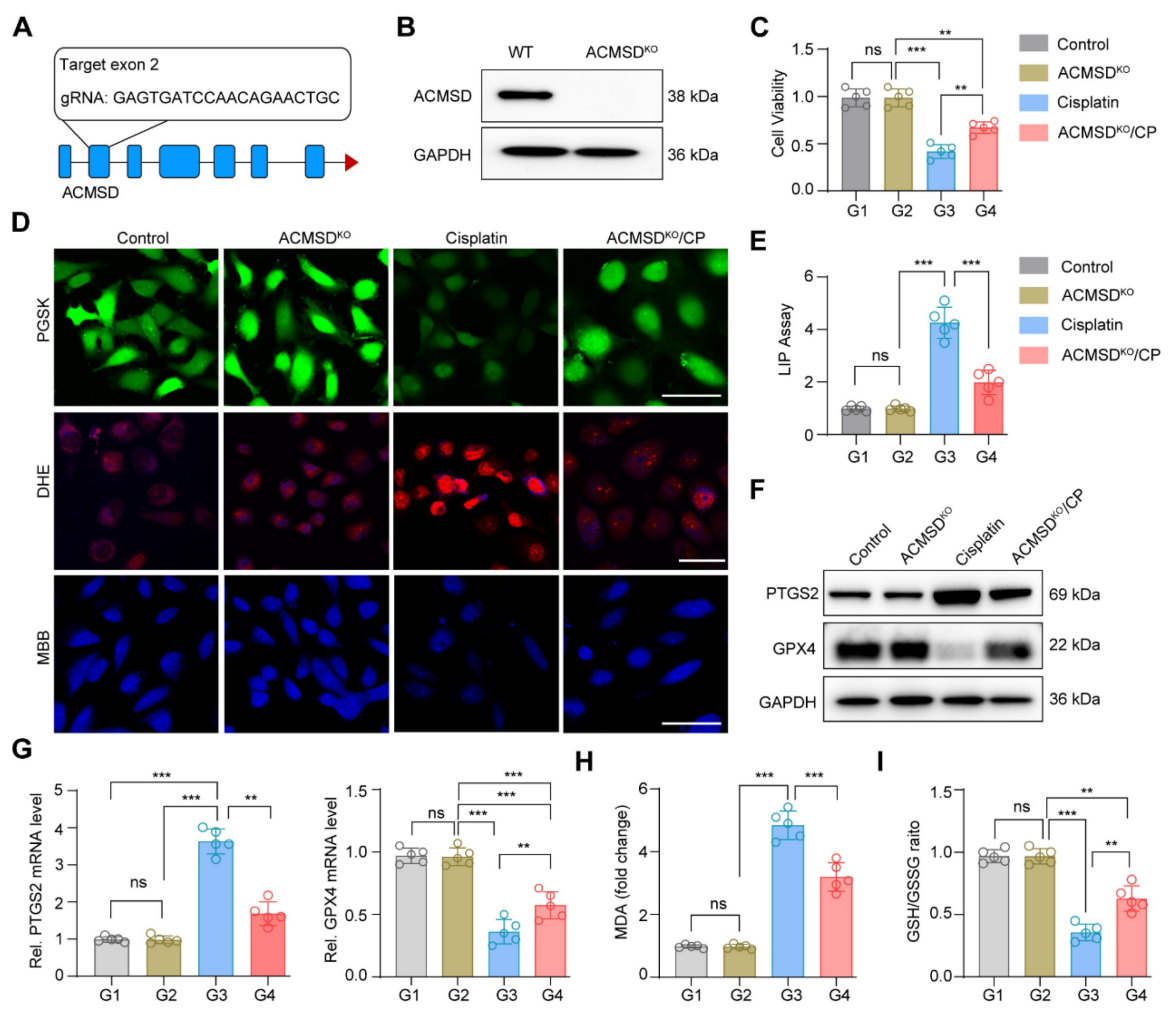
** Knockout of ACMSD alleviate CP-induced ferroptosis *in vitro***. (**A**) The construction strategy of CRISPR-Cas9 guide RNAs for ACMSD. (**B**) The protein expression of ACMSD in HK2 cells with or without CRISPR/Cas9-mediated knockout of the genes. (**C**) Cell viability of wild type or ACMSD knockout HK2 cells after CP exposure for 24 h (*n* = 5). (**D**) The representative fluorescent staining of PGSK, DHE and MBB staining in wild type or ACMSD knockout HK2 cells after CP exposure. Scale bars: 200 μm. (**E**) The LIP assay in wild type or ACMSD knockout HK2 cells after CP exposure (*n* = 5). (**F**) The protein expression levels of PTGS2 and GPX4 in wild type or ACMSD knockout HK2 cells after CP exposure. (**G**) The mRNA expression of *PTGS2* and *GPX4* in wild type or ACMSD knockout HK2 cells after CP exposure (*n* = 5). (**H**) The corresponding statistics of MDA in wild type or ACMSD knockout HK2 cells after CP treatment (*n* = 5). (**I**) The quantitative analysis of GSH level in wild type or ACMSD knockout HK2 cells after CP treatment (*n* = 5). All data are expressed as the mean ± s.d. (*, *P* < 0.05; **,* P* < 0.01; ***, *P* < 0.001 by one-way ANOVA with Tukey's multiple comparison test).

Our previous study demonstrated that the ACMSD protein was upregulated in HK2 cells and renal tissues significantly in AKI 17. To further clarify the expression distribution of ACMSD in AKI renal tissues, we analyzed the single-cell RNA sequencing (scRNA-seq) database (GSE139506) with the clinically relevant unilateral ischemia-reperfusion murine model of AKI at day 0 (control) and day 1 (the AKI injury occurred) 34. The results demonstrated that *ACMSD* mRNA was uniformly expressed in different renal tissue cells in the control but particularly high expression in renal tubular epithelial cells on day 1 in AKI (Figure S1A). We also examined the single-cell RNA sequencing (scRNA-seq) database (GSE139506) for the cisplatin (CP)-induced AKI model at day 0 and day 1 (Figure S1B and C). Results consistently demonstrated that *ACMSD* mRNA was highly expressed in renal tubular epithelial cells on day 1 when AKI occurred (Figure S1D). Interestingly, after treatment with erastin (a ferroptosis inducer), the protein expression of ACMSD was also significantly increased in the HK2 cells. These results suggest that ACMSD plays a significant role in AKI progression, particularly in ferroptosis-mediated AKI (Figure S2A). To further clarify the role of ACMSD in contributing of ferroptosis in AKI, we performed CRISPR-Cas9 method to knock out ACMSD permanently in HK2 cells (Figure 2A). Western blot analysis demonstrated that the HK2 cells did not express ACMSD proteins after gene editing (Figure 2B and Figure S2B). We then used the cisplatin (CP)-treated the HK2 cells to mimic the damage of nephrogenic tubular cells during AKI and subsequently examined the influence of ACMSD knock out on the ferroptosis progression. Consistently, the knockout of ACMSD prominently rescued the cell viability of HK2 cells following CP exposure (Figure 2C), reduced the cellular free iron levels and lipid oxidation level when stained with the PGSK or DHE probe, and rescued the reduced glutathione level (Figure 2D), as well as decreased the labile iron level (Figure 2E). Moreover, the knockout of ACMSD downregulated the expression of PTGS2 and promoted the expression of GPX4 in CP-induced HK2 cells (Figure 2F, G and Figure S2C, D). Simultaneously, the knockout of ACMSD significantly reduced the level of lipid oxidation and increased the protective GSH/GSSG ratio in CP treated HK2 cells (Figure 2H, I). These results demonstrated that ACMSD plays an important regulatory role in promoting ferroptosis and was an effective therapeutic target for AKI ferroptosis.

### ACMSD knockout antagonizes mitochondrial damage during ferroptosis *in vitro*

**Figure 3:**
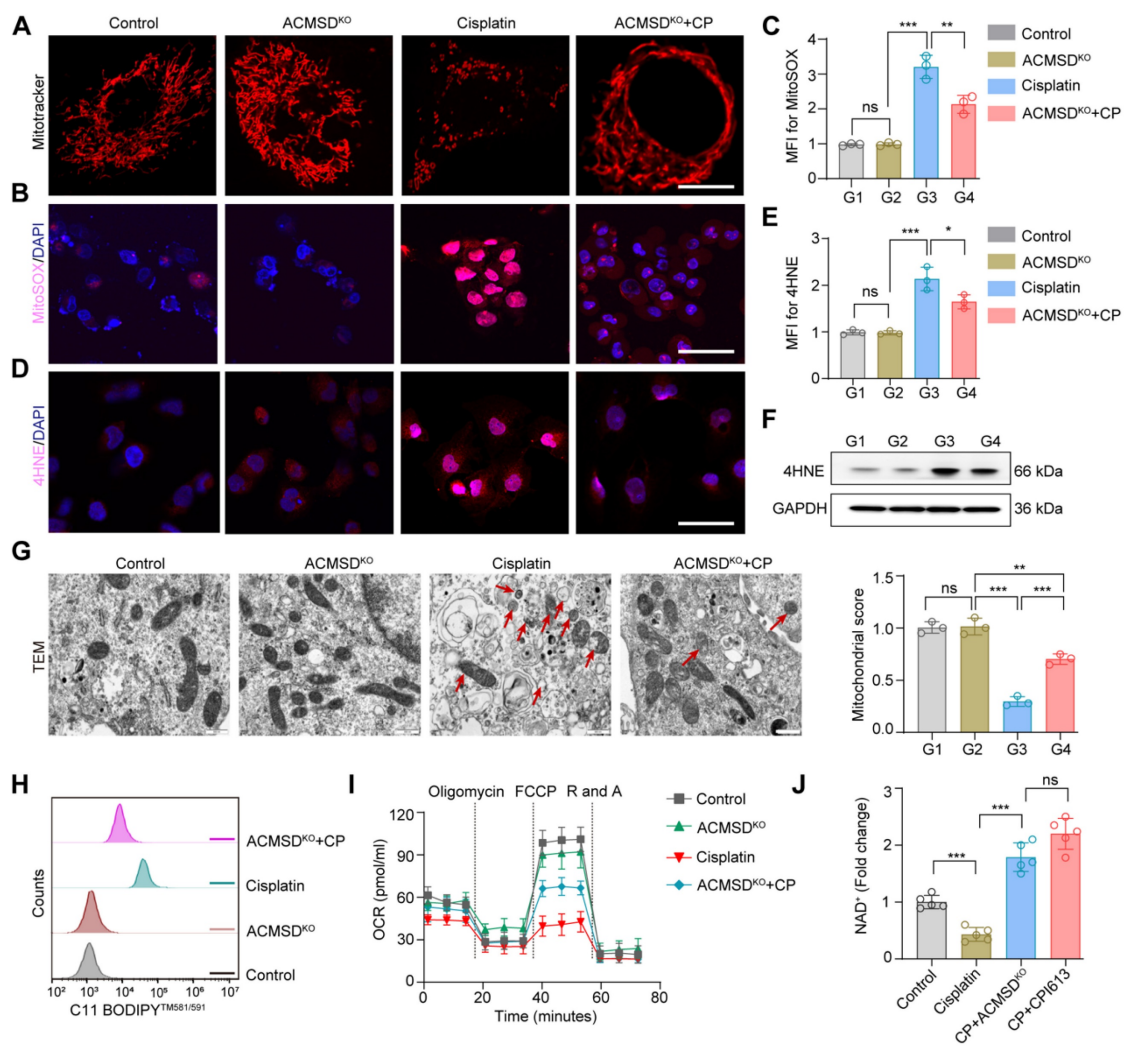
** ACMSD knockout antagonized mitochondrial damage during ferroptosis *in vitro***. (**A**) Representative MitoTracker staining images of wild-type and ACMSD knockout HK2 cells after CP exposure (*n* = 3). Scale bars: 10 μm. (**B, C**) MitoSOX assay in wild-type and ACMSD knockout HK2 cells after CP treatment. Scale bars: 100 μm. (**D, E**) 4HNE expression levels and corresponding statistical analysis in different groups (*n* = 3). Scale bars: 100 μm. (**F**) 4HNE protein expression levels in wild-type and ACMSD knockout HK2 cells after CP exposure. (**G**) The TEM images of mitochondria and completeness score from the wild type or ACMSD-knockout HK2 cells after CP exposure (*n* = 3). The red arrow represents the swollen mitochondria and ruptured membranes. Scale bars: 10 µm. (**H**) Flow cytometric analysis of C11 BODIPY^TM 581/591^ in different groups. (**I**) Mitochondrial respiration profiles in wild-type and ACMSD-knockout HK2 cells after different treatments for 24 h (*n* = 5). (**J**) Corresponding statistics for the NAD^+^ assay in wild-type and ACMSD-knockout HK2 cells after CP exposure (*n* = 5). All data are expressed as the mean ± s.d. Tukey-corrected two-way ANOVA was used for statistical analysis in (**I**). Tukey-corrected one-way ANOVA was used for statistical analysis in (**C**), (**E**), (**G**), and (**J**). (*, *P* < 0.05; **,* P* < 0.01; ***, *P* < 0.001).

Mitochondria are the main organelles that are structurally destroyed when cells are damaged by ferroptosis. To further explore the effects of ACMSD knockout on mitochondrial function during AKI, we characterized the morphology and function of mitochondria in wild-type and ACMSD-knockout HK2 cell when treated with CP. The results of MitoTracker staining showed the accumulation of fragmented mitochondria after CP treatment in the former cells, whereas mitochondria remained intact for longer and exhibited an even distribution in the latter cells (Figure 3A). In addition, assessment *via* the MitoSOX probe revealed that ACMSD knockout considerably reduced the accumulation of lipid ROS accumulation in mitochondria upon CP exposure (Figure 3B, C). Besides, both immunofluorescence and western blot analyses demonstrated a significant upregulation of the lipid peroxidation marker 4HNE in wild-type HK2 cells following CP exposure, whereas it was reduced in ACMSD knockout (Figure 3D-F and Figure S4A). Consistent with these results, the transmission electron microscopy (TEM) analysis showed that CP exposure induced mitochondrial damage in wild-type HK2 cells, as evidenced by the presence of a large number of swollen mitochondria along with the loss of cristae, with some also exhibiting ruptured membranes. Whereas in the ACMSD-knockout cells, the mitochondrial injury was considerably alleviated (Figure 3G). Additionally, the level of mitochondrial lipid oxidation was remarkably elevated in wild-type HK2 cells under CP exposure but notably reduced in ACMSD-knockout cells (Figure 3H). To further clarify whether the mitochondrial damage caused by CP exposure could be reversed, we used the JC-1 staining assay to determine the changes in mitochondrial membrane potential in wild-type and ACMSD-knockout HK2 cells after CP treatment. The results showed that ACMSD knockout relieved the CP-induced depolarization of mitochondrial membrane potential (Figure S3), and rescued the CP-induced mitochondrial damage and energy supply (Figure 3I). These results indicated that ACMSD knockout could eliminate the effects of AKI primarily by alleviating ROS-induced mitochondrial damage. NAD^+^ is a competitive metabolic substrate for ACMSD and the TCA cycle metabolism, reflects the potential of cells to resist aging and oxidative stress 16. To further clarify whether ACMSD knockout inhibited the TCA cycle and increased the NAD^+^ production, we examined the levels of cellular NAD^+^ and the main products of the TCA cycle in wild-type and ACMSD-knockout HK2 cells after CP treatment, as well as the TCA cycle-specific agonist CPI613 exposure. The results showed that the NAD^+^ level was considerably reduced during AKI, however ACMSD knockout halted such decrease, which was similar to the effect of the TCA cycle-specific agonists (Figure 3J). Concurrently, ACMSD knockout considerably inhibited the CP-induced TCA cycle progression and decreased the levels of alpha ketoglutaric acid (α-KG), succinic acid (SUC) and acetyl coenzyme A (CoA) (Figure S4B-D). These results indicated that knockout of ACMSD alleviated the injury caused by ferroptosis to renal tubular epithelial cells by inhibiting the TCA cycle, which is substantially active during this process.

### Preparation and characterization of ROS-responsive CVs targeting ACMSD

**Figure 4:**
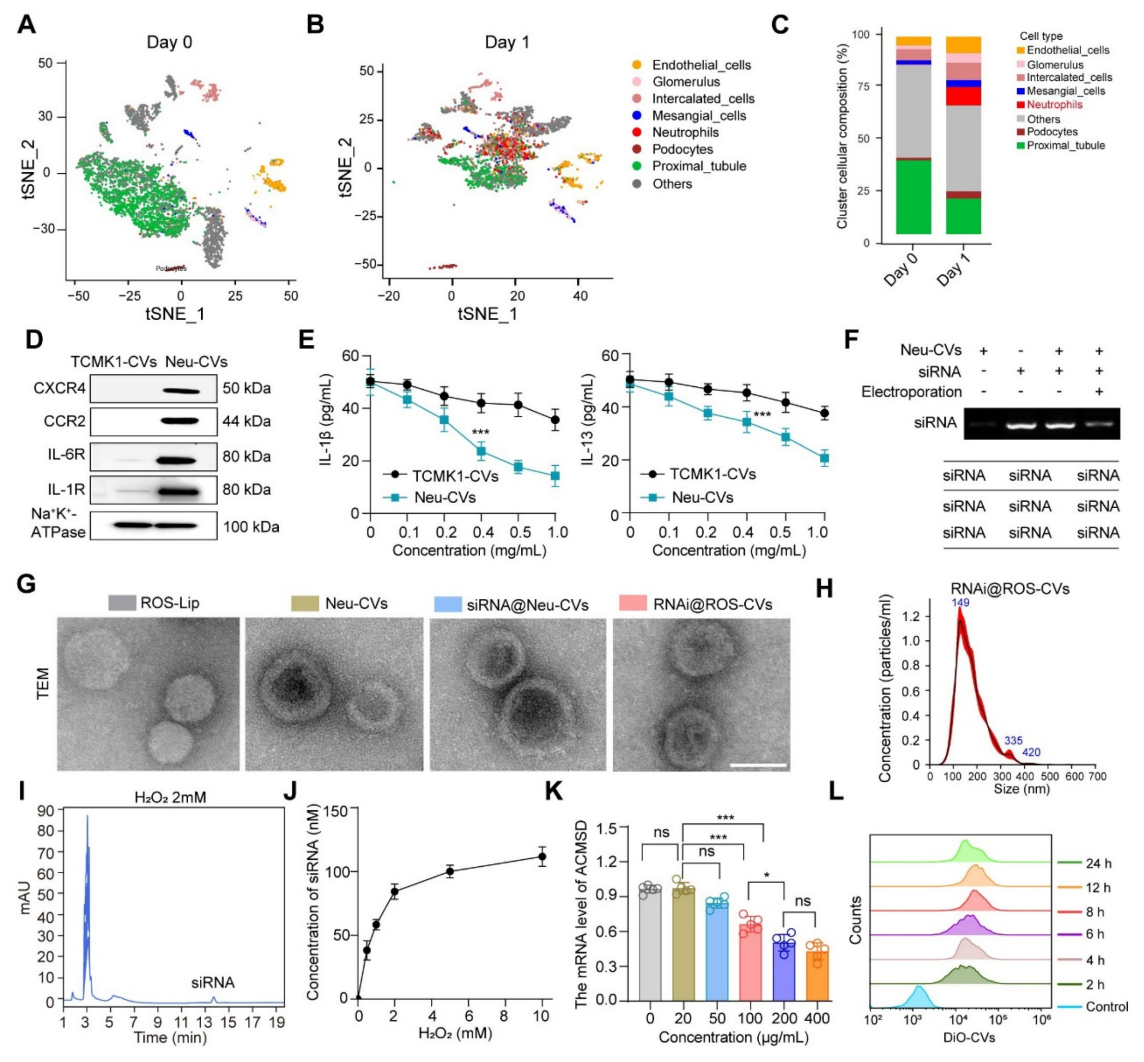
** Preparation and characterization of ROS-responsive CVs targeting ACMSD**. (**A, B**) UMAP plots showing the renal cell populations in the control on day 0 (**A**) and granulocyte populations on day 1 based on the scRNA-seq data for AKI mice kidney tissue. (**C**) The statistics of single-cell clustering of AKI kidneys on days 0 and 1. (**D**) The stable presence of proteins for chemokine and inflammatory receptors on the surface of neutrophil-derived CVs or TCMK1 derived CVs detected by western blot. (**E**) The adsorption ability of Neu-CVs to adsorb inflammatory factors *in vitro*. (**F**) The electric transfer condition of Neu-CVs for loading siRNA. (**G, H**) The TEM images of the ROS-Lip, Neu-CVs, RNAi@ROS-CVs, and RNAi@ROS-CVs and results of nanoparticle tracking analysis (NTA). Scale bars: 200 nm. (**I**) The release ability of siRNA in RNAi@ROS-CVs detected by HPLC in the ROS condition. (**J**) The curve of siRNA released by RNAi@ROS-CVs with the change of ROS concentration detected by HPLC. (**K**) The RNAi@ROS-CVs mediated ACMSD knockdown *in vitro*. (**L**) The endocytosis of HK2 cells on RNAi@ROS-CVs detected by flow cytometry at different exposure times. All data are expressed as the mean ± s.d. Tukey-corrected two-way ANOVA was used for statistical analysis in (**E**) Tukey-corrected one-way ANOVA was used for statistical analysis in (**K**). Two-tailed unpaired t test was used for statistical analysis in (**E**). (*, *P* < 0.05; **,* P* < 0.01; ***, *P* < 0.001).

Given the pivotal role of ACMSD in regulating ferroptosis and alleviating AKI, we hypothesized that the targeted knockdown of ACMSD expression *in vivo* may offer a promising strategy for AKI therapy. Previous studies have shown that AKI is accompanied by a significant inflammatory response, especially in the early stage, when neutrophils instantly migrate to the injured renal tubules 35. We analyzed the scRNA-seq database (GSE139506/GSE220675) for a murine model of AKI on days 0 and 1 after AKI onset. The results showed that neutrophils were considerably increased in renal tissues on day 1, which was not observed in normal renal tissues on day 0 (Figure 4A-C, Figure S1C and S5A). Encouraged by these results, we anticipated that the neutrophil-derived CVs (Neu-CVs) could exert an innate targeting effect against AKI and represent a promising avenue for drug delivery platforms for inflammatory diseases. Using the isolation process previously reported 21, we isolated neutrophils from peripheral blood samples collected from inflamed mice and obtained nanoscale CVs by consecutive sonication of membranes, followed by extrusion through nanopores using a mini extruder (Figure S5B). Given that Neu-CVs serve as prospectively biocompatible-carriers for functional biomolecules with a high loading capacity for inflammatory chemotaxis, we characterized the functional molecules and biological functions of Neu-CVs. The western blot results confirmed the presence of abundant chemokine receptors and inflammatory factor receptors on the surface of Neu-CVs, including CXCR4, CCR2, IL-6R and IL-1R, which predictably means that Neu-CVs have the biological function of targeting inflammation and adsorbing cytokines (Figure 4D and Figure S5C). Relying on these cytokine receptors, Neu-CVs efficiently adsorb inflammatory cytokines (Figure 4E), including IL-1β and IL-13, in a dose-dependent manner, indicating their potential moderating effect on the inflammatory response. Subsequently, the ACMSD-knockdown siRNA was loaded into the Neu-CVs by electroporation to finally obtain the AKI-targeting nanomedicine for ferroptosis intervention (Figure 4F). The residual content of siRNA in the transfection supernatant was determined by high-performance liquid chromatography (HPLC) and DNA gel electrophoresis experiments, which also aided in confirming the siRNA encapsulation efficiency. The encapsulation efficiency of siRNA was ~10% (1 mg protein concentration of CVs load 100 nM siRNA) (Figure S6).

Considering the rapid onset of AKI and its debilitating effects, therapeutic intervention drugs must also be rapidly released to block the disease process. To this end, we constructed ROS-responsive liposomes (ROS-Lip) and, using a phospholipid membrane fusion and extrusion strategy, we fused the ROS-responsive element to siRNA-loaded Neu-CVs to obtain an AKI-targeting and ROS-responsive drug (RNAi@ROS-CVs) (Figure S7A and B). We characterized the ROS-Lip, Neu-CVs, and engineered CVs loaded with siRNA *via* nanoparticle tracking analysis (NTA) and TEM visualization. The results showed the engineered CVs remained as round lipid droplets similar to the Neu-CVs and had with a similar average size of 150 nm (Figure 4G, H). We investigated the particle size of the RNAi@ROS-CVs NPs redispersed after long-term storage and found that no significant degradation or agglomeration occurred during the long-term storage (Figure S8A and B), and the morphology of NPs displayed a regular circular lipid structure (Figure S8C). Concurrently, using the HPLC method and standard curve, we confirmed that the siRNA in the RNAi@ROS-CVs platform could be released efficiently in the ROS condition, and its abundance depended on H_2_O_2_ concentration (Figure 4I, J). In addition, the mRNA expression of *ACMSD* in HK2 cells could be knocked down effectively in a dose-dependent manner of RNAi@ROS-CVs NPs *in vitro* (Figure 4K). Concurrently, the dye-labeled RNAi@ROS-CVs could be phagocytized by HK2 cells in a time-dependent manner, with the phagocytosis effect reaching its peak at 12 h (Figure 4L). Overall, these results confirmed the successful development of neutrophil-derived ROS-responsive CVs that can be used for targeted intervention to block ACMSD and inhibit ferroptosis.

### Nanoparticle-mediated ACMSD knockdown inhibits ferroptosis progression *in vitro*

**Figure 5:**
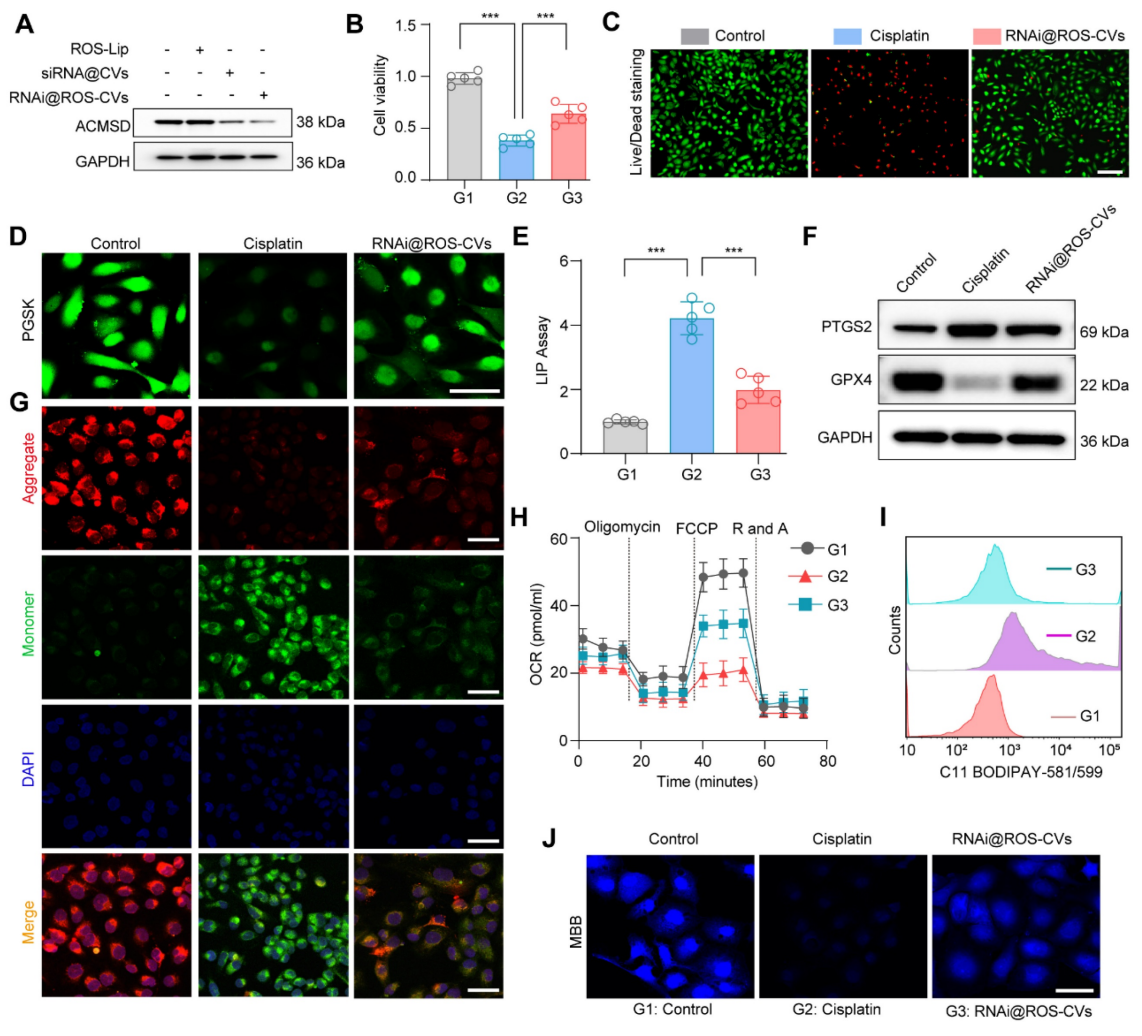
** The nanoparticles mediated knockdown of ACMSD inhibits ferroptosis progression and alleviated cell injury *in vitro***. (**A**) The protein expression of ACMSD in HK2 cells after treatment with RNAi@ROS-CVs. (**B**) Cell viability in wild type and RNAi@ROS-CVs-pretreated HK2 cells under CP exposure. (*n* = 5). (**C**) Live/Dead staining for wild type or RNAi@ROS-CV-pretreated HK2 cells following CP exposure. Scale bars: 200 µm. (**D**) Representative fluorescent staining of PGSK in wild type or RNAi@ROS-CVs-pretreated HK2 cells when CP treatment. Scale bars: 100 µm. (**E**) The LIP assay in wild type or RNAi@ROS-CV-pretreated HK2 cells when CP treatment (*n* = 5). (**F**) The protein expression of PTGS2 in different groups. (**G**) The representative JC-1 staining for mitochondrial membrane potential in wild type or RNAi@ROS-CV-pretreated HK2 cells. Scale bars: 50 μm. (**H**) Mitochondrial respiration profiles in different groups (*n* = 5). Scale bars: 10 µm. (**I**) Flow cytometric analysis of C11 BODIPY^TM 581/591^ in the different groups. (**J**) The representative fluorescent staining of MBB in wild type or RNAi@ROS-CVs-pretreated HK2 cells following CP treatment. Scale bars: 50 µm. All data are expressed as the mean ± s.d. Tukey-corrected two-way ANOVA were used for statistical analysis in (**H**). Tukey-corrected one-way ANOVA was used for statistical analysis in (**B**) (**E**). (*, *P* < 0.05; **,* P* < 0.01; ***, *P* < 0.001).

Next, we further evaluated the efficiency of RNAi@ROS-CVs NPs for knocking down ACMSD *in vitro*, and the rescue effect for ferroptosis. The results showed that the RNAi@ROS-CVs NPs effectively knocked down the expression of ACMSD in HK2 cells, and the knockdown efficiency for ROS-responsive CVs was higher than ROS non-responsive CVs (Figure 5A and Figure S9A). In addition, the ACMSD knockdown mediated by the CVs effectively reduced the mortality of CP-treated HK2 cell thereby increasing their survival (Figure 5B, C). The content of divalent iron and intracellular free iron in PGSK-labeled cells was also decreased, which surely led to iron-dependent ferroptosis alleviation (Figure 5D, E). Concurrently, treatment with RNAi@ROS-CVs considerably decreased the expression of PTGS2 (the main driver maker of ferroptosis), and increased the mRNA and protein expression of GPX4 after CP exposure (Figure S9B-E and Figure 5F). In addition, the RNAi@ROS-CVs-mediated ACMSD knockdown simultaneously reduced the CP-induced depolarization of the mitochondrial membrane potential, rescued the mitochondrial function, and increased the energy supply capacity of HK2 cells (Figure 5G, H). Consistently, ACMSD knockdown also reduced the level of lipid oxidation and increased that of GSH in HK2 cells under CP treatment (Figure 5I, J). Because Neu-CVs could adsorb the inflammatory factors (Figure 4E), to exclude the contribution of this intrinsic anti-inflammatory property to the observed inhibition of ferroptosis, we used the Neu-CVs vector to treat HK2 cells that had undergone ferroptosis. Results showed that the treatment with Neu-CVs did not reduce the expression of PTGS2, nor did it increase the expression of GPX4 (Figure S10). This finding indicates that ferroptosis alleviation in RNAi@ROS-CVs NPs was caused by the inhibition of ACMSD. All above results indicated that the ROS-responsive Neu-CVs loaded with siRNA could alleviate the ferroptosis process and rescue cell injury *in vitro* by effectively reducing the protein expression of ACMSD.

### Targeting and therapeutic effects of RNAi@ROS-CVs *in vivo*

**Figure 6:**
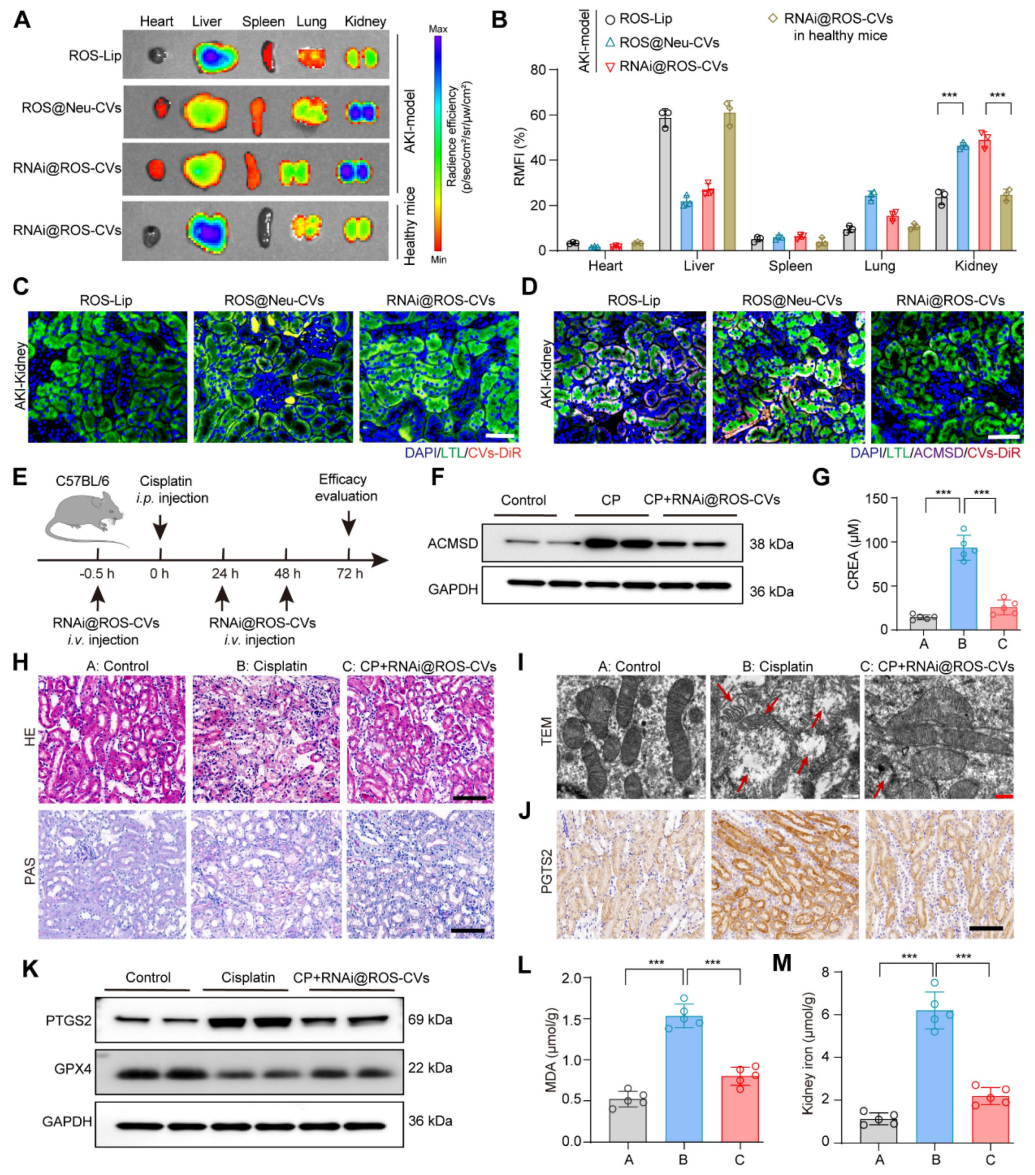
** Targeting and therapeutic effects of RNAi@ROS-CVs *in vivo***. (**A, B**) Fluorescence imaging using the IVIS system and corresponding fluorescence intensities for major organs after *i.v.* injection of fluorescently labeled nanoparticles in AKI kidney and healthy kidneys after 24 h (*n* = 3). (**C**) The fluorescently labeled RNAi@ROS-CVs in the interior of renal tubular epithelial cells. Scale bars: 100 µm. (**D**) The colocalization of RNAi@ROS-CVs, antibody labeled-LTL and ACMSD in renal tubular epithelial cells. Scale bars: 100 µm. (**E**) Schematic of the experimental design for AKI model* in vivo*. (**F**) The protein expression of ACMSD in the kidney after RNAi@ROS-CVs treatment. (**G**) The level of serum creatinine after different treatments (*n* = 5). (**H**) The H&E and PAS staining of kidney sections in AKI after different treatments. Scale bars: 100 µm. (**I**) The TEM images of mitochondria from the kidney section of different groups. The red arrow represents the swollen mitochondria and ruptured membranes. Scale bars: 5 µm. (**J**) Protein expression of PTGS2 in the kidney tissue among different groups determined by IHC staining. Scale bars: 100 µm. (**K**) Protein expression of PTGS2 and GPX4 in the kidney tissue among different groups. (**L**) (**M**) The levels of MDA and free iron in the kidneys in different groups (*n* = 5). All data are expressed as the mean ± s.d. Tukey-corrected two-way ANOVA was used for statistical analysis in (**B**). Tukey-corrected one-way ANOVA was used for statistical analysis in (**G**) (**K**) (**L**). (*, *P* < 0.05; **, *P* < 0.01; ***, *P* < 0.001).

Due to the chemotactic and migratory properties of neutrophils in inflammatory lesions, neutrophil-derived CVs are usually well able to target these type of lesions. Thus, we hypothesized that the Neu@ROS-CVs may help increase the levels of tissue retention and targeting efficacy for renal lesions with better therapeutic effects. To this end, we labeled the ROS-Lip, Neu-CVs and RNAi@ROS-CVs with DiR and injected the NPs intravenously into C57BL/6 mice with CP-induced AKI or healthy control. At different timepoints after injection, the fluorescence intensities of major organs were detected to reveal the accumulation of reagents. The results showed that ROS-Lip mainly accumulated in the liver after intravenous injection, whereas ROS-responsive Neu-CVs were mainly deposited in the injured kidneys (Figure 6A). Consistent with these results, the RNAi@ROS-CVs also demonstrated a good targeting effect on injured kidneys, whereas its targeted effect on healthy kidneys was mild (Figure 6A, B). Furthermore, the RNAi@ROS-CVs was rapidly accumulated the kidney of AKI mice within 2 h of administration, peaked at ~8 h, and remained in the AKI kidney until 48 h (Figure S11A, B), suggesting that the CV-modified NPs hve excellent targeting efficiency on the injured kidney. Fluorescent staining of treated kidney tubules revealed that the Neu-CVs were mainly localized in the proximal renal tubule (Figure 6C and Figure S12), and exhibited good levels of colocalization with a lectin from *Lotus tetragonolobus* (LTL), a marker protein of renal proximal tubules (Figure 6D and Figure S13). Besides, KIM-1 Amb was also used to mark the injured renal tubules and track the subcellular localization of RNAi@ROS-CVs NPs in AKI kidneys. The results demonstrated that RNAi@ROS-CVs were primarily colocalized in the renal tubules that expressed KIM-1 and mediated the downregulation of expression of KIM-1 (Figure S14). All these results demonstrated the successful establishment of an* in vivo* delivery system for targeting inflammation-related diseases and may mediated the intervention therapy.

Encouraged by the promising lesion-targeting action of RNAi@ROS-CVs validated in injured kidney, we set out to demonstrate their therapeutic effects on AKI kidney using the CP-exposed mice model. C57BL/6 mice were intravenously pretreated with RNAi@ROS-CVs and subsequently exposed with CP intraperitoneal injection to induce AKI. Subsequently, the mice were treated with RNAi@ROS-CVs once again at 24 h and 48 h after CP injection (Figure 6E). Not surprisingly, the NPs considerably suppressed the ACMSD protein expression because of targeted siRNA delivery (Figure 6F and Figure S15A). In addition, the creatinine levels of the AKI mice were reduced (Figure 5G) and the degree of kidney injury was considerably rescued when ACMSD was targeted block (Figure 6H). Strikingly, compared with other treatment groups, the CP-exposed mice injected with RNAi@ROS-CVs exhibited a normal mitochondrial morphology (Figure 5I), decreased the protein expression of PTGS2, 4HNE (Figure 6I, J, and Figure S15B, C, S16), and increased expression of GPX4 and FTH1 (Figure 6J, K, and Figure S15D, S17). Furthermore, the levels of lipid oxidation and free iron in AKI kidneys were also substantially decreased under the treatments of RNAi@ROS-CVs NPs (Figure 6L and 6M), and the indicators of inflammation such as IL-6, IL-1, CXCL-2, CXCL-8 and CXCL-12 were decreased (Figure S18A-E), as well as the mRNA level of injury-related indicators such as *KIM-1* and neutrophil gelatinase-associated lipocalin (*NGAL*) (Figure S18F, G). Consistently, the levels of blood urea nitrogen (BUN) and urine protein in AKI mice were also decreased simultaneously after the ACMSD was knocked down (Figure S19A, B). These results indicated that our blocking strategy targeting for ACMSD is effective for preventing AKI and will has the potential to be used in multilesion models.

### The RNAi@ROS-CVs relieve the IR-induced AKI *in vivo*

**Figure 7:**
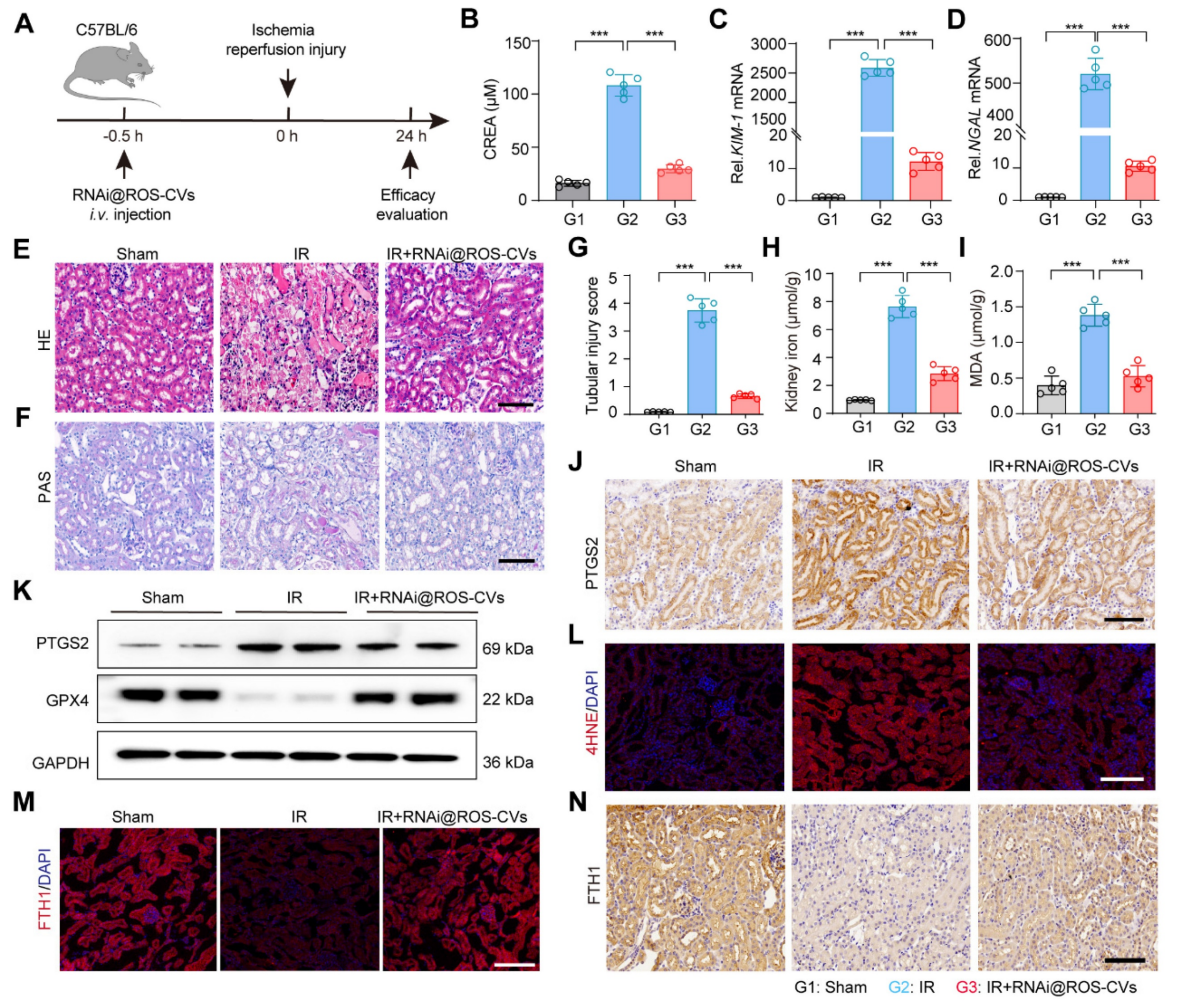
** RNAi@ROS-CVs relieve the IR induced AKI *in vivo*.** (**A**) Schematic of the experimental design for the IR-induced AKI model* in vivo*. (**B**) The level of serum creatinine in different groups (*n* = 5). (**C, D**) The mRNA expression levels of *KIM-1* and *NGAL* in the kidney tissue in different treatment groups (*n* = 5). (**E, F**) The H&E and PAS staining of kidney sections in AKI-affected mice after different treatments. Scale bars: 100 µm. (**G**) The renal injury score for AKI after different treatments (*n* = 10). (**H, I**) The levels of free iron and MDA in the kidney of different groups (*n* = 5). (**J, K**) The protein expression of PTGS2 in the kidney tissue in different groups. (**L**) The protein expression of 4HNE in the kidney tissue in different groups. Scale bars: 100 µm. (**M, N**) The expression of FTH1 protein in the kidney tissue in different groups. Scale bars: 100 µm. All data are expressed as the mean ± s.d. (*, *P* < 0.05; **, *P* < 0.01; ***, *P* < 0.001 by one-way ANOVA with Tukey's multiple comparison test).

Next, using an ischemia reperfusion (IR) model, we further elucidated the therapeutic effect of RNAi@ROS-CVs on AKI mice that were intravenously pretreated with RNAi@ROS-CVs and subsequently exposed in IR. Then, the rescue effect on the injured kidney was assessed during the subsequent 24 h. (Figure 7A). Consistent with the results of CP-mediated AKI, the ACMSD protein level in the renal tissue was significantly increased after IR-mediated ferroptosis, whereas its expression could be effectively inhibited by pretreatment with RNAi@ROS-CVs NPs (Figure S20A). Furthermore, the levels of creatinine in IR mice were decreased (Figure 7B), the mRNA levels of *KIM-1* and *NGAL* in AKI kidney were downregulated (Figure 7C, D), the degree of renal injury was considerably rescued, as well as the glycogen deposition and the injury score when pretreatment with RNAi@ROS-CVs (Figure 7E-G). The levels of serum free iron and the extent of lipid peroxidation in the renal tissues were also significantly decreased upon ACMSD knockdown (Figure 7H, I). Moreover, the expression of the PTGS2 and 4HNE in kidney were downregulated (Figure 7L, Figure S20E and Figure S21), whereas that of GPX4 (Figure 7J, K and Figure S20B-D) and FTH1 were significantly up-regulated after RNAi@ROS-CVs treatment (Figure 7M-N). Consistently, the level of BUN and urine protein were also decreased simultaneously after pretreatment with NPs in mice with IR-mediated AKI mice (Figure S22A and B). Interestingly, after treating the mice with folic acid to induce AKI, the ACMSD protein expression level also increased significantly in the kidney tissues, indicating that ACMSD is a crucial molecular target in driving kidney injury and has the significance as a universal therapeutic target (Figure S22C).

Considering the potential safety concerns of introducing large amounts of nucleic acids and the side effects of off-target effects *in vivo*, we conducted an acute toxicity test for RNAi@ROS-CVs in mice. Intravenous injection with RNAi@ROS-CVs at a dose of 5 mg/kg for 7 consecutive days did not cause disorders or the pathogeny structure injuries of main organs, including liver, kidney, and heart (Figure S23), nor did it affect the body weight of the mice blood cells or blood biochemistry (Figure S24). Collectively, RNAi@ROS-CVs were highly effective in knocking down ACMSD *in vivo*, successfully targeting the renal injury lesions, inhibiting ferroptosis, and alleviating AKI with negligible safety impacts.

## Discussion

Ferroptosis is a nonapoptotic cell death mechanism characterized by the iron-dependent peroxidation of membrane lipids 36, 37. The kidney exhibits more vulnerable to oxidative stress and ferroptotic processes due to its high rate of oxygen consumption, excessive catalytic activities by iron-containing proteins, and exposure to concentrated redox-active compounds 38-40. Ferroptosis is a feature of AKI that leads to the deterioration of kidney function and is implicated in various etiologies 6, 41. Viewing ferroptosis from a cell biological perspective advances our understanding of how to best promote or interfere with this process by modulating the function of different structures within the cell, as well as assisting in the development of promising potent drugs targeting ferroptosis 42. Herein, we demonstrated that ACMSD promotes AKI by stimulating TCA cycle and exacerbating ferroptosis process, its knockdown *in vitro* blocks this process and reverses kidney damage. Using the targeting principle of neutrophil-derived CVs, we designed a multifunctional controlled-release drug delivery platform to carry nucleic-acid molecules targeting for ACMSD, which can block ACMSD expression in renal tubular epithelial cells and rescue AKI *in vitro* and *in vivo* without detection of biological toxicity. This universal drug delivery and controlled-release system may represent a precision therapeutic strategy for inflammatory diseases, not limited to kidney disease.

Notably, the present studies suggested that the metabolic mediation regulated by ACMSD, particularly for NAD^+^ metabolism, is associated with organ injury risks and may be linked to the pathogenesis of organ risk *via* TCA cycle-related pathways, including a direct enhancement in ROS production and the suppression of mitochondrial function 12, 43. The tissue-specific expression of ACMSD in the kidney and liver provides an important opportunity as it allows selective targeting or blocking of these tissues without causing side effects. In fact, a sound rationale for targeting ACMSD has emerged for the treatment of disease in recent years. Some efficacies were reported with methodologies developed to target pathogenic ACMSD using TES1025, an effective and selective inhibitor of human, nevertheless, these strategies are sometimes restricted by issues such as non-specific targeting, inefficient delivery or safety concerns 12. Therefore, exploring a general platform or strategy to deliver RNAi or TES1025 to lesions accurately and synchronously targeted rapid drug release will be valuable.

Unlike other synthetic nanoparticles, CVs are highly safe *in vivo* as they are consisted of lipids, proteins, and nucleic acids 23. Owing to their inherent adaptability for disease targeting, and ease of genetic, chemical, or physical engineering, CVs may become a typical platform for the integration of multivalent functional biomolecules and targeted drug delivery 44, 45. The aforementioned properties highly facilitate the targeted application of CVs *in vivo* because, small molecule drugs in these conditions usually require prolonged circulation and increased lesion targeting to improve therapeutic efficacy and reduce side effects. In particular, CVs contain transmembrane and membrane anchored proteins (such as CD47) that likely protect cells or drugs from phagocytosis through CD47-SIRPα “don't eat me” signal 46-48. In addition to retaining the chemotactic properties of neutrophils, Neu-CVs also have unique biological functions 49, 50, such as immunosuppressive and tissue repair functions. The uptake of Neu-CVs by macrophages could increase the level of itaconate in them, thereby suppressing hyperinflammation 51. Therefore, Neu-CVs are naturally ideal drug delivery vectors for targeted therapy of inflammatory diseases 52. Owing to their properties, they have an enhanced ability to deliver RNAi and specifically target ACMSD in lesion of AKI.

Herein, we used Neu-CVs for this purpose, with the aim of enhancing the therapeutic effect by targeting lesions and reducing cytokine levels, as well as controlling drug release. We expect that this study could combine the characteristics of AKI-targeting therapeutic nucleic-acid drugs and cellular-derived CVs to provide a foundation for the continued development of multifunctional CVs. Thus, it will become a new type of nanomedicine for targeted therapy for inflammatory diseases in the future.

## Supplementary Material

Supplementary figures and table.
