# Supplementary material for: ROS-responsive cellular vesicles with ferroptosis-targeting siACMSD delivery for acute kidney injury therapy
Source: Theranostics. 2026 Jan 1;16(4):1941–58. doi: 10.7150/thno.119667 (PMC12680598; doi:10.7150/thno.119667)
Supplement: Supplementary file 1 — Supplementary figures and table. [file thnov16p1941s1.pdf]

# ROS-responsive cellular vesicles with ferroptosis-targeting siACMSD delivery for acute kidney injury therapy

Yunjing Zhang[1†], Qing Deng[1†], Yangtao Xu[2†], Wei Wu[3], Tian Wu[1], Jia Huang[1], Yugang Hu[1], Weiqiang Lin[4*], Ximing Xu[2*]and Jicheng Wu[2*]

[1]Department of Ultrasound, Renmin Hospital of Wuhan University, Wuhan 430060, China.

[2]Cancer Center, Renmin Hospital of Wuhan University, Wuhan 430060, China.

[3]Department of Intensive Care Unit, Renmin Hospital of Wuhan University, Wuhan 430060, China.

[4]Department of Nephrology, Center for Regeneration and Aging Medicine, The Fourth Affiliated Hospital of School of Medicine and International School of Medicine, International Institutes of Medicine, Zhejiang University, Yiwu 322000, China.

*Correspondence to: W.L., wlin@zju.edu.cn; X.X., doctorxu120@aliyun.com; J.W., jicheng wu@whu.edu.cn

[†] These authors contributed equally to this work.

**Table of Contents:**

- Supplementary Figures
- Supplementary Table

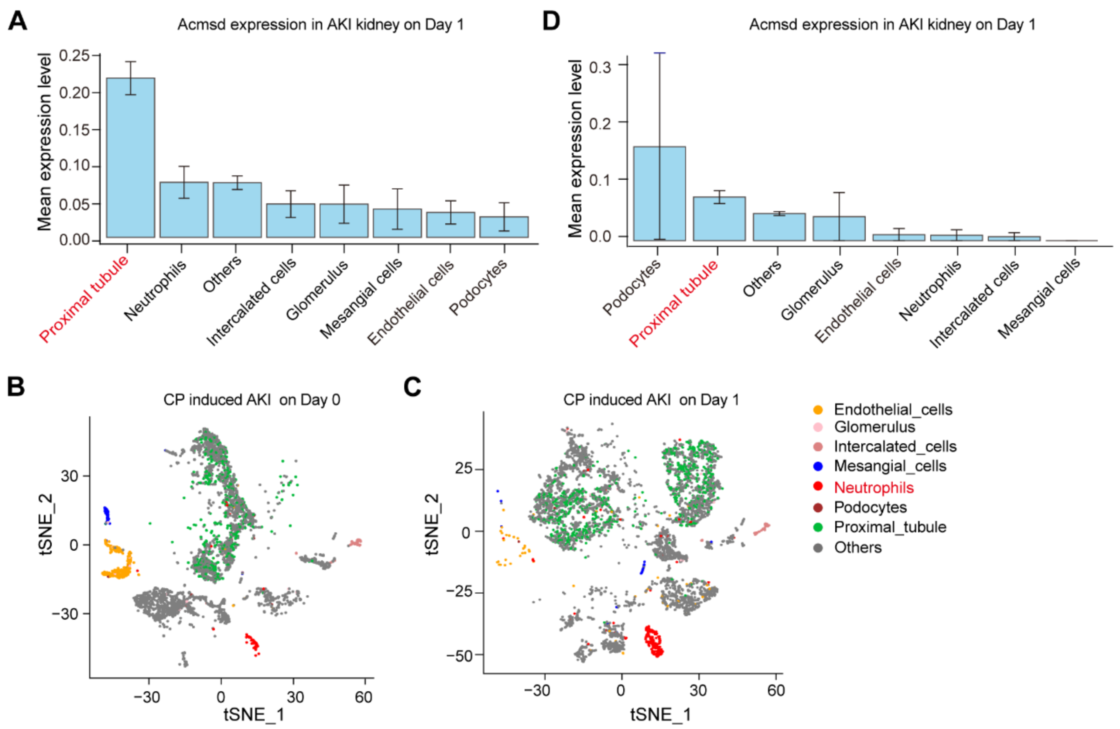


**Figure S1. The expression of ACMSD is significantly up-regulated in renal tubular epithelial cells during AKI**. (**A**) The distribution of *ACMSD* mRNA expression in renal tissue in UIR induced AKI on day 1. (**B**, **C**) UMAP plots showing the renal cell populations in the control on day 0 and AKI on day 1 based on the scRNA-seq data for CP-induced AKI kidney tissue. (**D**) The distribution of *ACMSD* mRNA expression in renal tissue in CP-induced AKI on day 1.

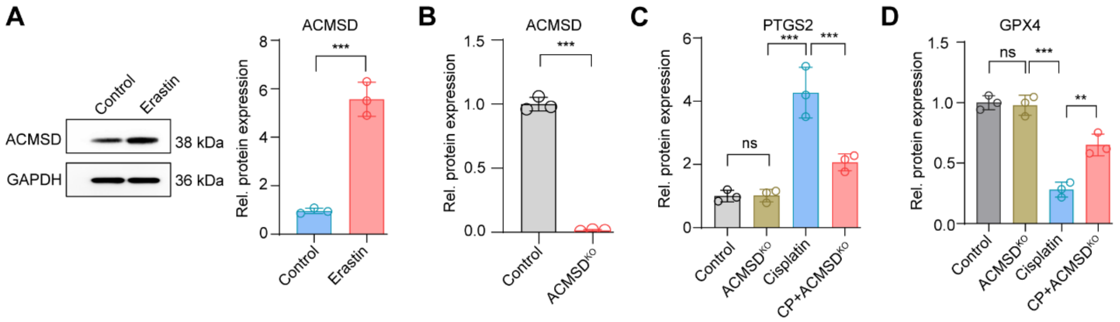


**Figure S2. Knockout of ACMSD alleviate CP-induced ferroptosis *in vitro*. (A)** The protein expression and statistical analysis of ACMSD in HK2 cells after erastin treatment ($n = 3$). **(B)** The protein expression levels of ACMSD in wild type or ACMSD knockout HK2 cells after CP exposure ($n = 3$). **(C)** The statistical analysis of protein expression for ACMSD in wild type or ACMSD knockout HK2 cells after CP exposure ($n = 3$). **(D)** The statistical analysis of protein expression for GPX4 in wild type or ACMSD knockout HK2 cells after CP exposure ($n = 3$). Data are presented as means ± sd. Tukey-corrected two-way ANOVA was used for statistical analysis in (I). Two-tailed unpaired t test was used for statistical analysis in (**A**) (**B**). Tukey-corrected one-way ANOVA was used for statistical analysis in (**C**) (**D**). ns: not significant, $^{***}P < 0.001$.

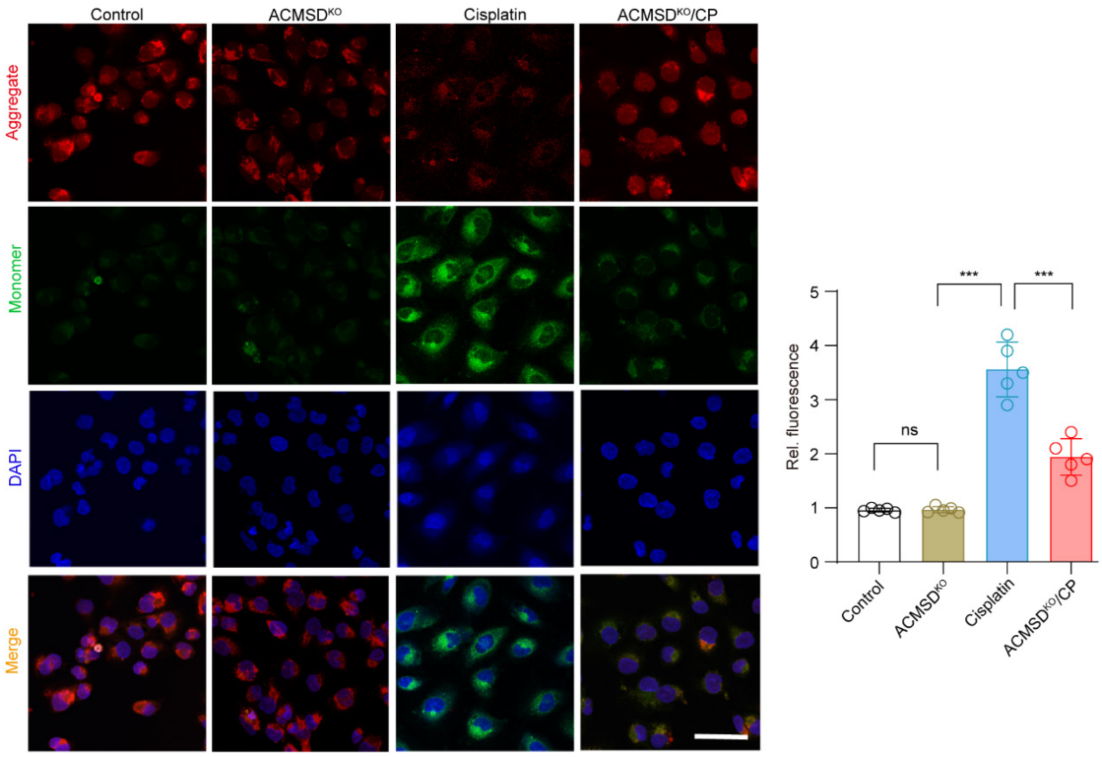


**Figure S3. ACMSD KO reverses CP-induced mitochondrial potential depolarization in HK2 cells**. The mitochondrial membrane potential was measured by immunofluorescence in Control, ACMSD$^{KO}$, CP, ACMSD$^{KO}$ + CP groups ($n = 3$). Scale bars: 200 μm. Data are presented as means ± sd. Tukey-corrected one-way ANOVA was used for statistical analysis. ns: not significant, $^{***}P < 0.001$.

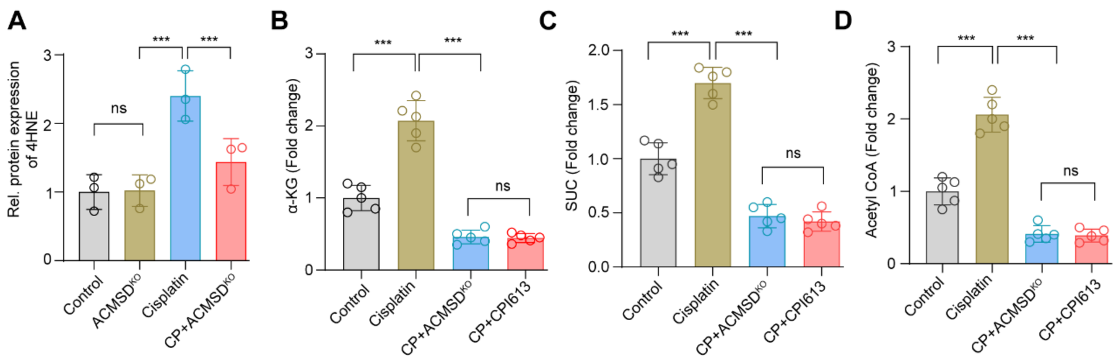


**Figure S4. ACMSD KO inhibits the increase of TCA cycle during AKI *in vitro*.** (**A**) The statistical analysis of protein expression for 4HNE in wild type or ACMSD knockout HK2 cells after CP exposure ($n = 3$). (**B**-**D**) The metabolites of the TCA cycle were measured in different treatment groups ($n = 3$). Data are presented as means ± sd. Tukey-corrected one-way ANOVA was used for statistical analysis. ns: not significant, $^{***}P < 0.001$.

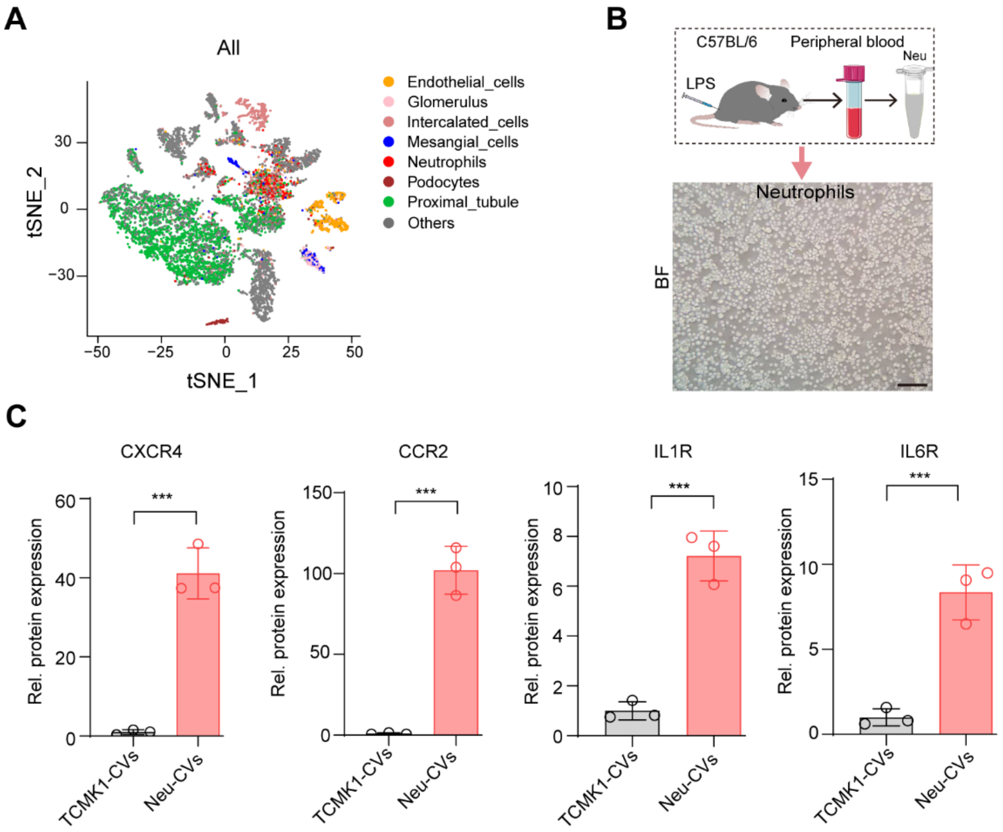


**Figure S5. The stable expression of proteins of chemokine and inflammatory receptors on the surface of neutrophils derived CVs**. (**A**) The UMAP plots of the general cell classification in the scRNA-seq data for AKI mice kidney tissue. (**B**) Overview of the process for neutrophils extraction from mouse peripheral blood and the morphology under microscopy. Scale bars: 100 μm. (**C**) The statistical analysis of protein expression for chemokine and inflammatory receptors on the surface of neutrophils derived CVs ($n$ = 3). Data are presented as means ± sd. Tukey-corrected one-way ANOVA was used for statistical analysis. ns: not significant, $^{***}P < 0.001$. Two-tailed unpaired t test was used for statistical analysis.

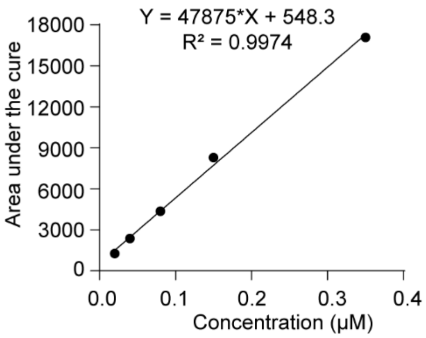


**Fig. S6.** The area under the curve of the chromatograms of free siRNA was plotted against concentration to make a standard curve by HPLC.

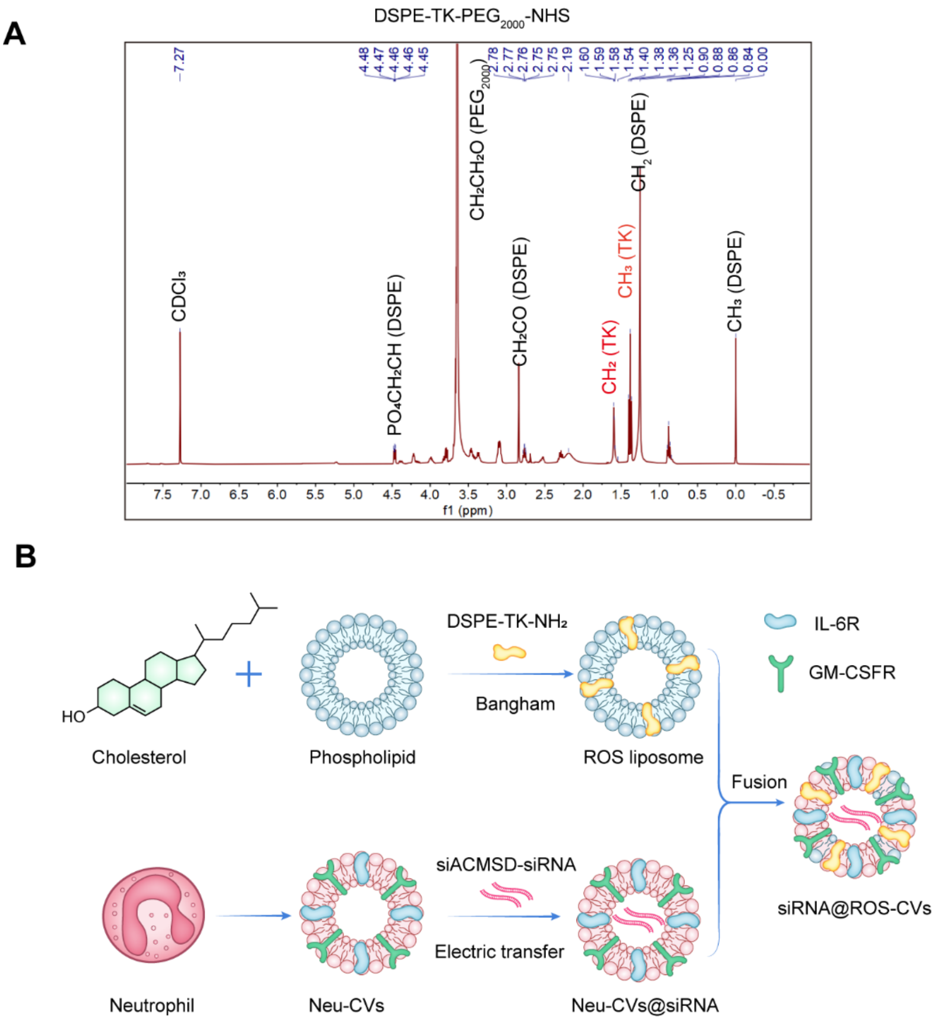


**Figure S7. Strategies for the preparation of ROS responsive CVs**. (**A**) The $^{1}$H-NMR spectra of the ROS responsive DSPE-TK-PEG2000. (**B**) Construction strategy of RNAi@ROS-CVs nanoparticles targeting for ACMSD.

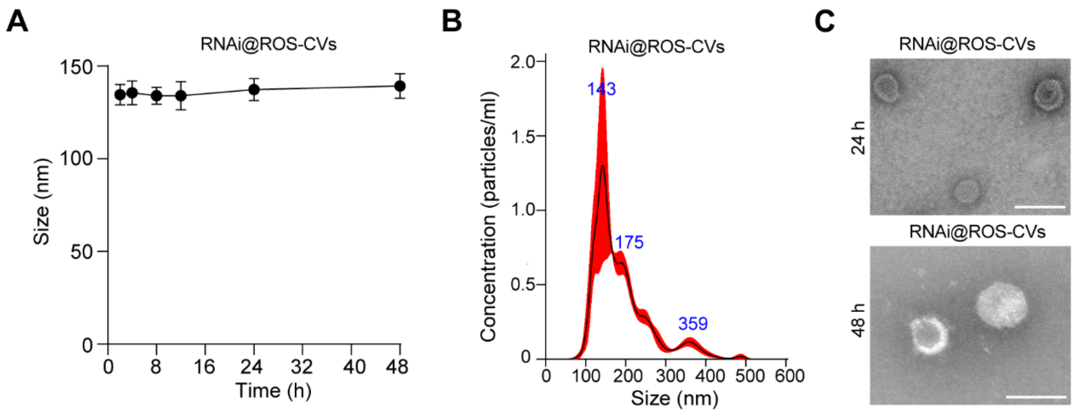


**Figure S8. Preparation and characterization of RNAi@ROS-CV NPs**. (**A**) The size distribution of RNAi@ROS-CV NPs on 7 consecutive days measured by NTA ($n$ = 3). (**B**) The particle size of the RNAi@ROS-CVs after being stationary for 48 h detected by NTA. (**C**) The representative TEM images of RNAi@ROS-CVs at 24 h and 48 h. Scale bars: 200 nm.

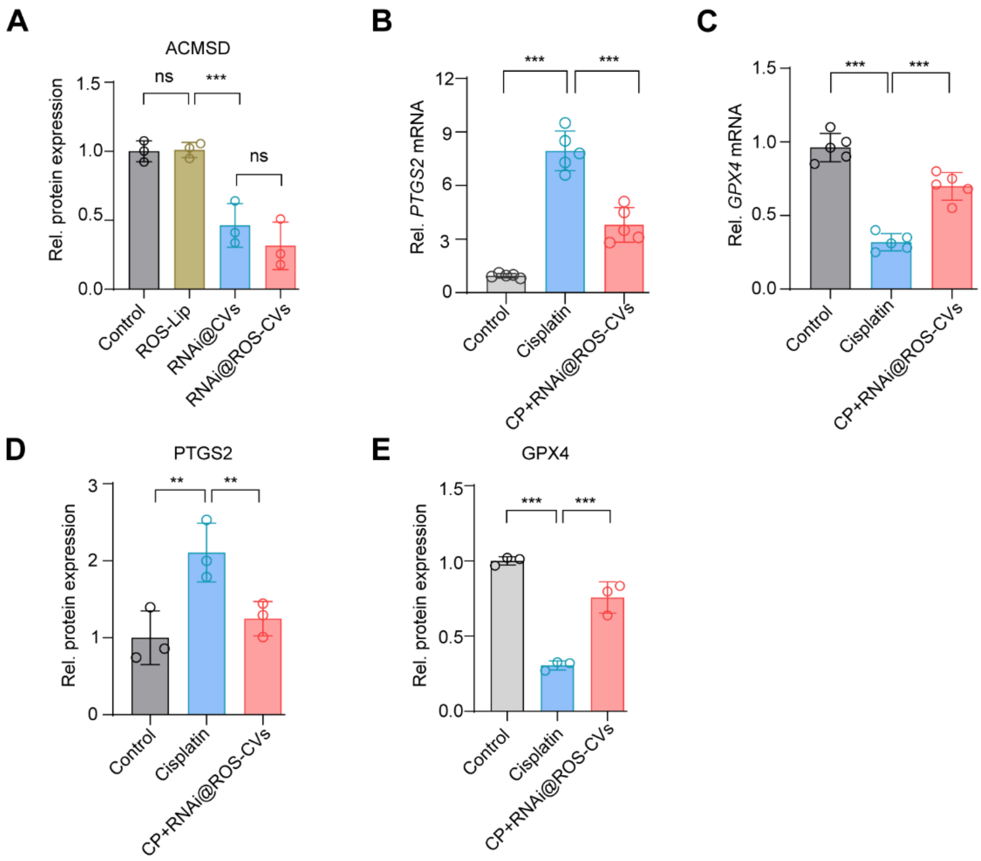


**Figure S9. RNAi@ROS-CV NPs mediated ACMSD Knockout alleviate CP-induced ferroptosis *in vitro*.** (**A**) The statistic analysis of protein expression for HK-2 cells after different treatment ($n$ = 3). (**B**) The mRNA expression of *PTGS2* in HK-2 cells after different treatment ($n$ = 5). (**C**) The mRNA expression of *GPX4* in HK-2 cells after different treatment. (**D**) The statistic analysis of PTGS2 protein expression for HK-2 cells after different treatment ($n$ = 3). (**E**) The statistic analysis of GPX4 protein expression for HK-2 cells after different treatment ($n$ = 3). Data were presented as means ± sd. ns: not significant, $^{**}P < 0.01$, $^{***}P < 0.001$. One-way ANOVA with Tukey's multiple comparison test was used for statistical analysis.

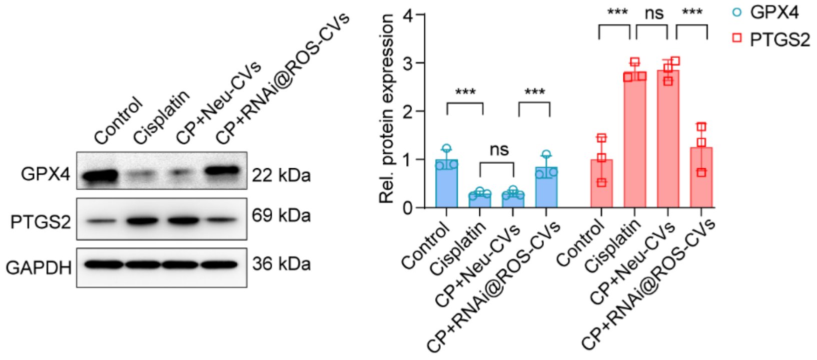


**Figure S10. It is the RNAi@ROS-CVs, not Neu-CVs, alleviates CP-induced ferroptosis**. The protein expression and statistic analysis of GPX4, and PTGS2 in HK-2 cells after different treatment (*n* = 3 for each group). Data were presented as means ± sd. Tukey-corrected two-way ANOVA were used for statistical analysis.

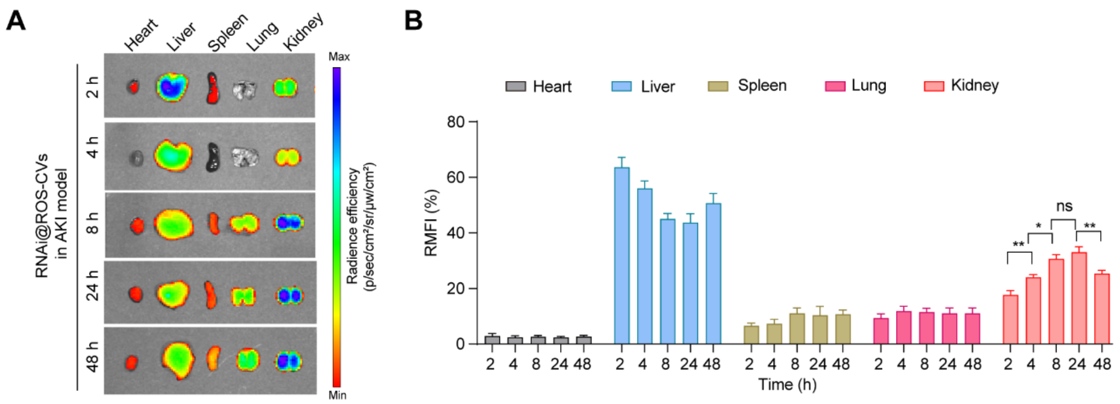


**Figure S11. The targeting effects of RNAi@ROS-CVs in AKI kidney *in vivo*.** (**A, B**) The fluorescence imaging and corresponding fluorescence intensities of major organs after *i.v.* injection of fluorescently labeled RNAi@ROS-CVs nanoparticles in AKI mice for different time ($n = 3$). Data were presented as means ± sd. Tukey-corrected two-way ANOVA were used for statistical analysis.

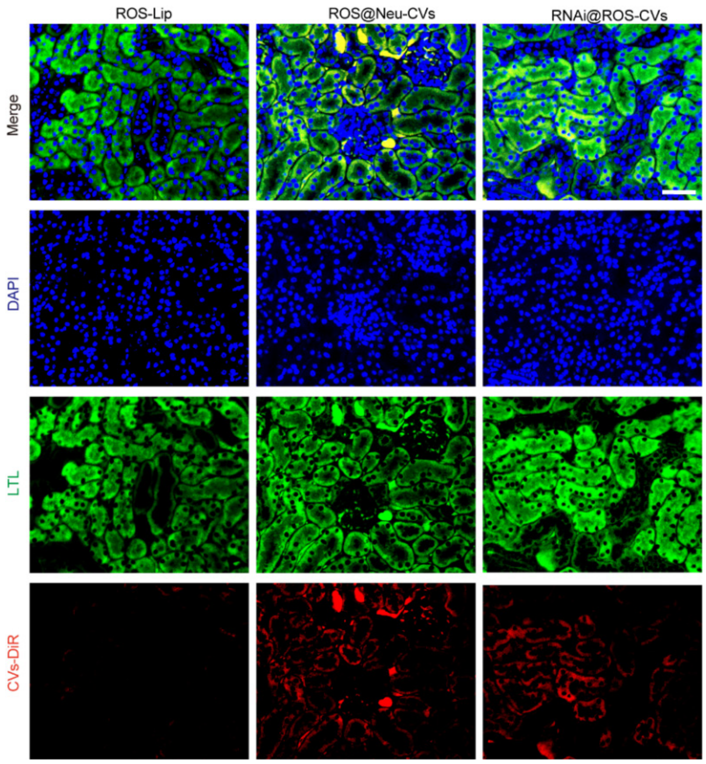


**Figure S12. The targeting effects of RNAi@ROS-CVs in AKI kidney *in vivo*.** The colocalization of DIR labeled RNAi@ROS-CVs and antibody labeled-LTL in renal tubular epithelial cells. Scale bars: 100 μm.

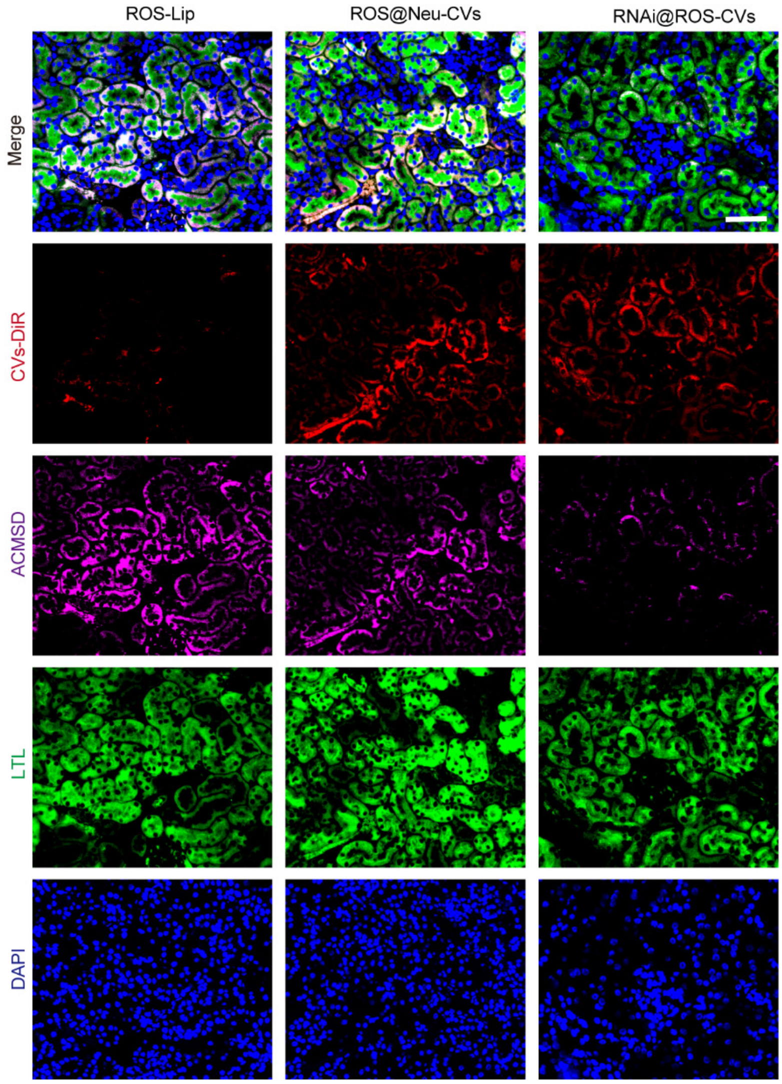


**Figure S13. The RNAi@ROS-CVs NPs mediated ACMSD knockdown effect in AKI kidney *in vivo***. The colocalization of DIR labeled RNAi@ROS-CVs, antibody labeled-LTL and ACMSD in renal tubular epithelial cells. Scale bars: 100 μm.

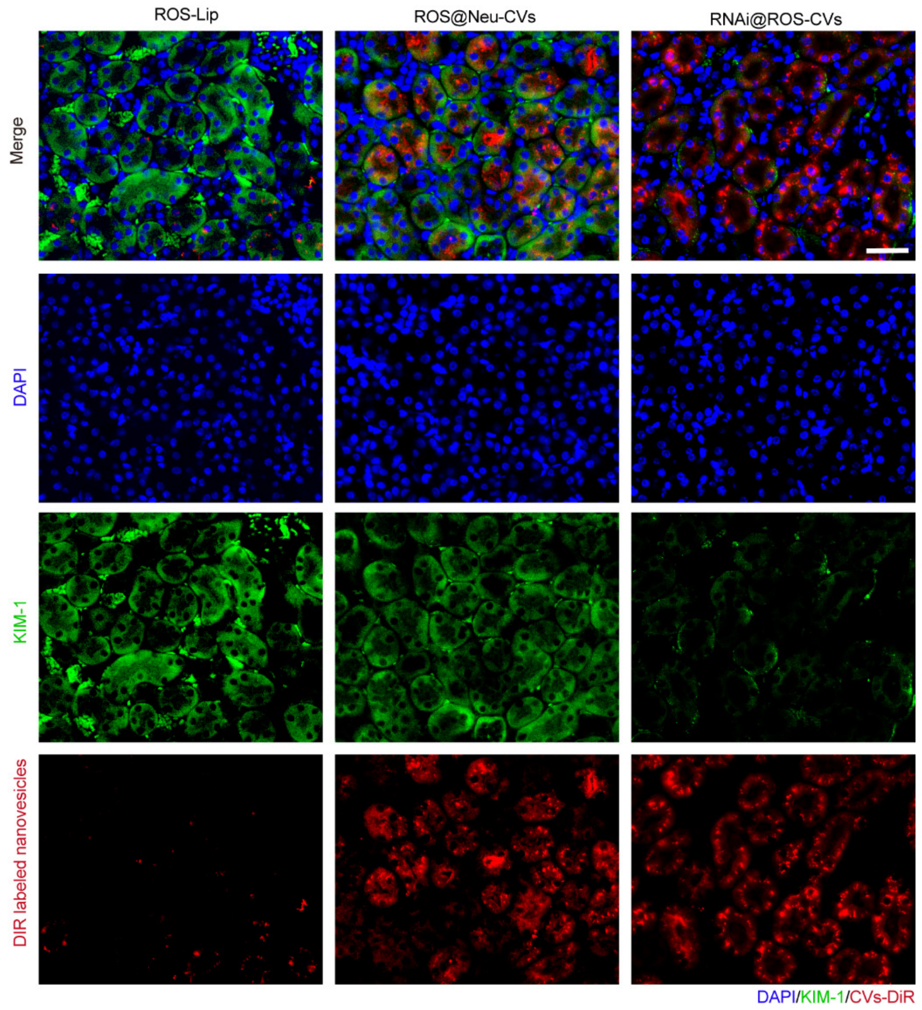


**Figure S14. The targeting effects of RNAi@ROS-CVs in AKI kidney *in vivo*.** The colocalization of DIR labeled RNAi@ROS-CVs and antibody labeled KIM-1 in renal tubular epithelial cells. Scale bars: 100 µm..

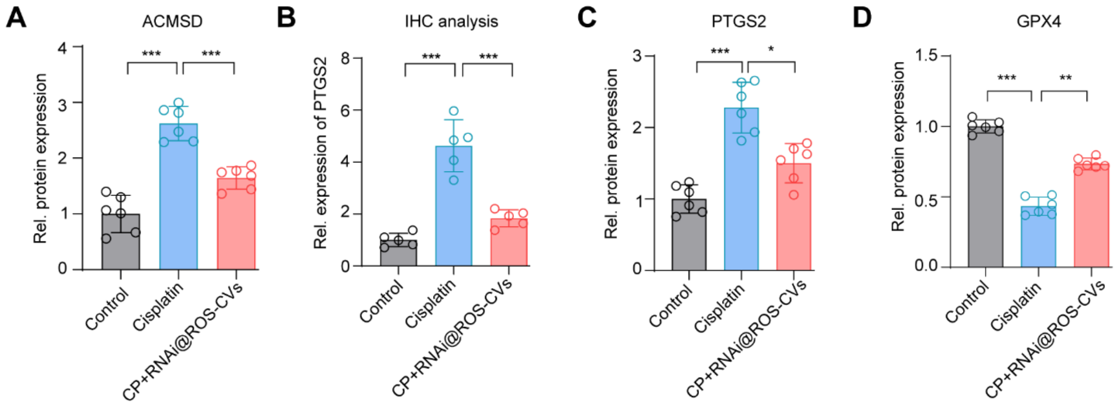


**Figure S15. RNAi@ROS-CVs mediated ACMSD Knockout alleviate CP-induced ferroptosis *in vivo*.** (**A**) The statistical analysis of protein expression for ACMSD in AKI kidney after different treatment ($n$ = 6). (**B**) The statistical analysis of protein expression for ACMSD in AKI kidney after different treatment when detect by IHC (6). (**C**) The statistical analysis of protein expression for PTGS2 in AKI kidney after different treatment ($n$ = 6). (**D**) The statistical analysis of protein expression for GPX4 in AKI kidney after different treatment ($n$ = 6). Data were presented as means ± sd. ns: not significant, **P < 0.01, ***P < 0.001. One-way ANOVA with Tukey' s multiple comparison test was used for statistical analysis..

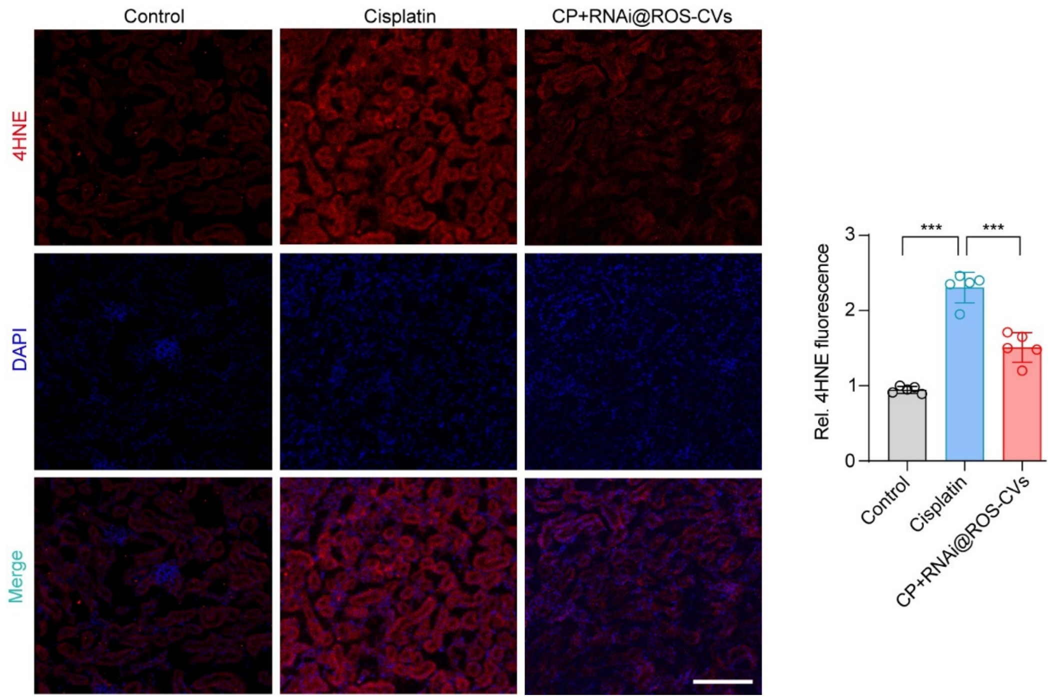


**Figure S16.** RNAi@ROS-CVs ameliorates overexpression of 4HNE in CP-induced AKI model. Representative immunofluorescence images of 4HNE in different groups and quantification of positive areas (*n* = 5 per group). Scale bars, 50 µm. Data were presented as means ± SD. ($P < 0.001$ by one-way ANOVA with Tukey's multiple comparison test)

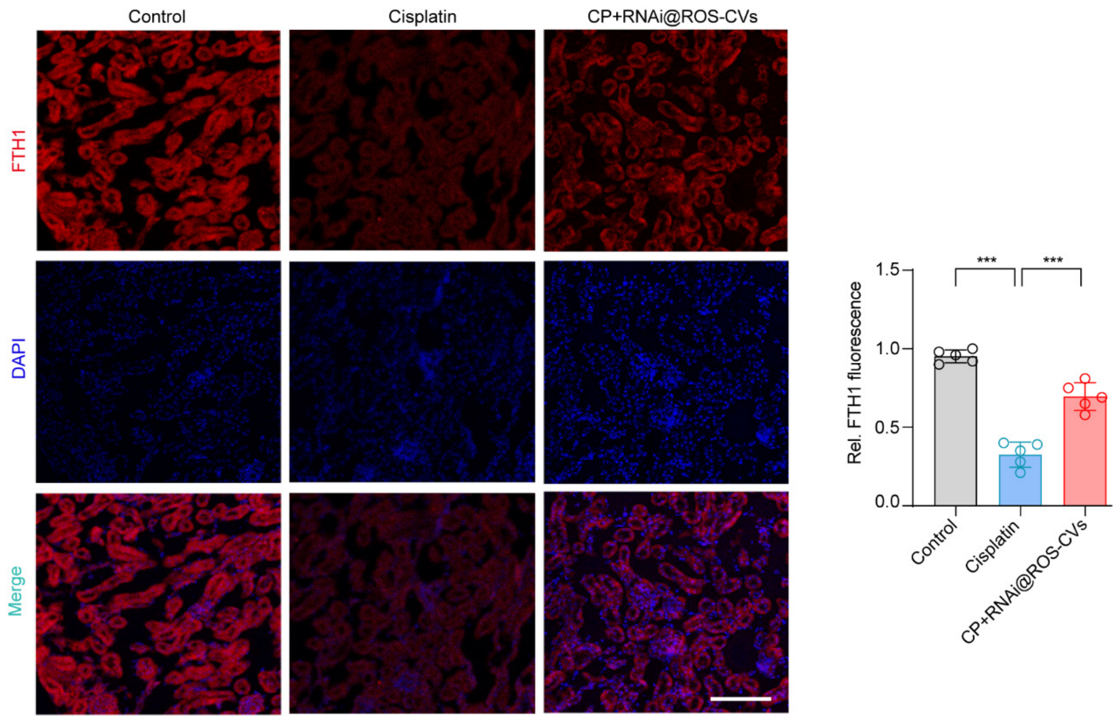


**Figure S17. RNAi@ROS-CVs reverses down-regulation of FTH1 in CP-induced AKI model**. Representative immunofluorescence images of FTH1 in different groups and quantification of positive areas ($n$ = 5 per group). Scale bars, 50 μm. Data were presented as means ± SD. ($^{***}P < 0.001$ by one-way ANOVA with Tukey's multiple comparison test).

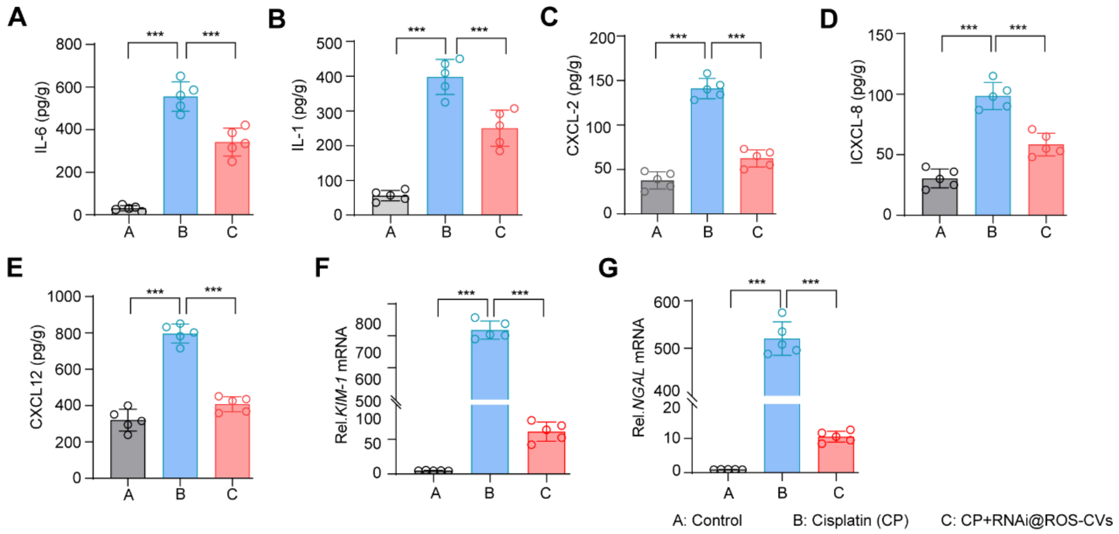


**Figure S18. The RNAi@ROS-CVs alleviates CP-induced ferroptosis and inflammation**. (**A-E**) The cytokines level of IL-6, IL-1 CXCL-2, CXCL-8 and CXCL-12 in AKI kidney after different treatment ($n$ = 5). (**F, G**) Quantification of *KIM-1* and *NGAL* mRNA in different groups ($n$ = 5). Data were presented as means ± sd. ($^{***}P <$ 0.001 by one-way ANOVA with Tukey's multiple comparison test)

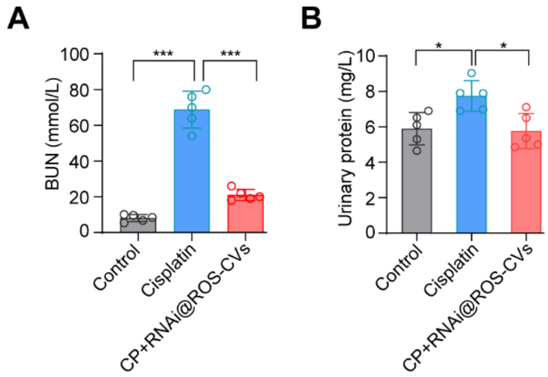


**Figure S19. The RNAi@ROS-CVs alleviates CP-induced kidney injury and the production of urine protein**. (**A**) The blood urea nitrogen (BUN) levels of AKI mice after different treatment ($n = 5$). (**B**) The urine protein levels of AKI mice after different treatment ($n = 5$). Data were presented as means ± sd. ($^{***}P < 0.001$ by one-way ANOVA with Tukey's multiple comparison test)

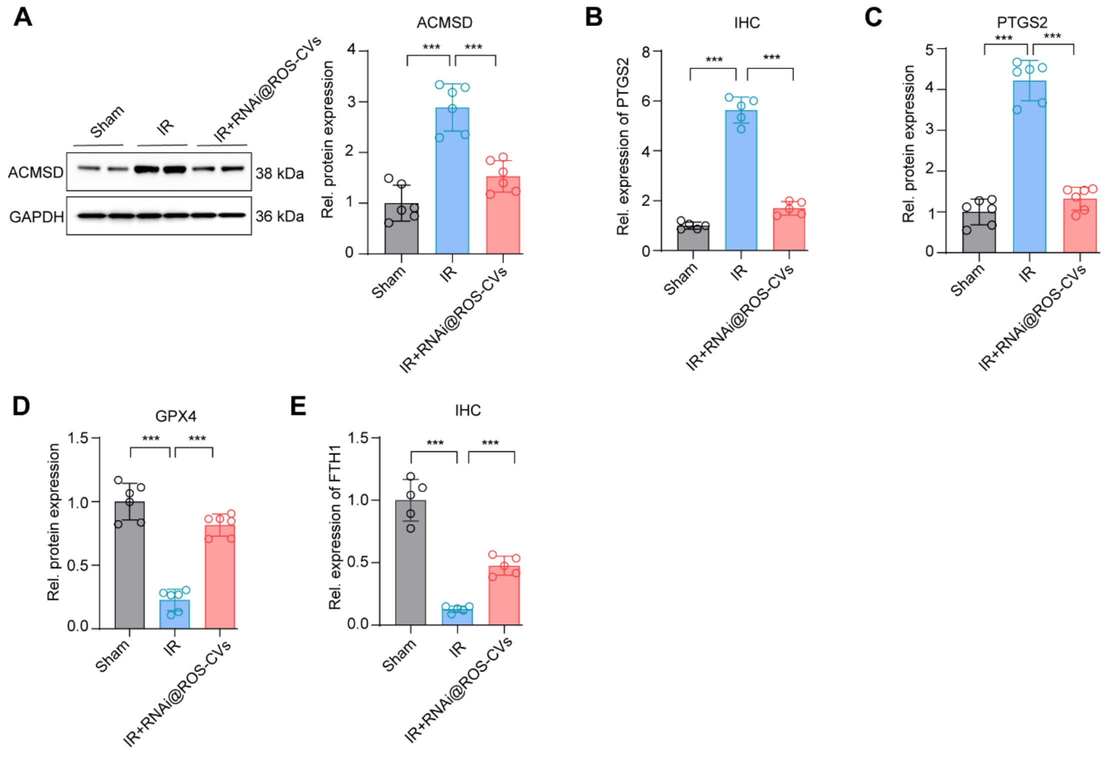


**Figure S20. The RNAi@ROS-CVs alleviates IR-induced ferroptosis**. (**A**) The protein expression and statistical analysis of ACMSD in mice kidney after different treatment ($n$ = 6). (**B**) Quantification of protein expression for PTGS2 in mice kidney detected by IHC after different treatment ($n$ = 5). (**C**) The statistical analysis of ACMSD in mice kidney when detected by western blot after different treatment ($n$ = 5). (**D**) The statistical analysis of GPX4 in mice kidney when detected by western blot after different treatment ($n$ = 5). (**E**) Quantification of protein expression for PTGS2 in mice kidney detected by IHC after different treatment ($n$ = 5). Data were presented as means ± sd. ($^{***}P < 0.001$ by one-way ANOVA with Tukey's multiple comparison test)

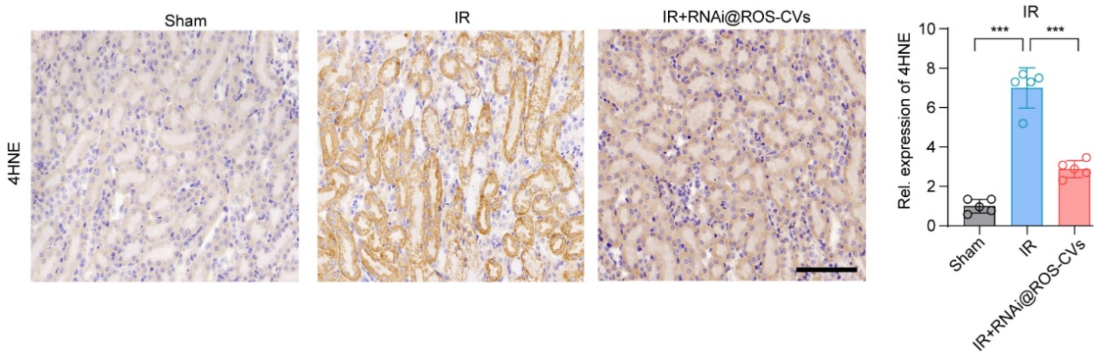


**Figure S21. RNAi@ROS-CVs alleviates IR-induced overexpression of 4HNE**. Representative photograph of IR-induced AKI tissues stained with 4HNE, the immunohistochemistry result showed that the increased expression of 4HNE induced by IR was partially decreased by RNAi@ROS-CVs. Scale bars, 100 μm.

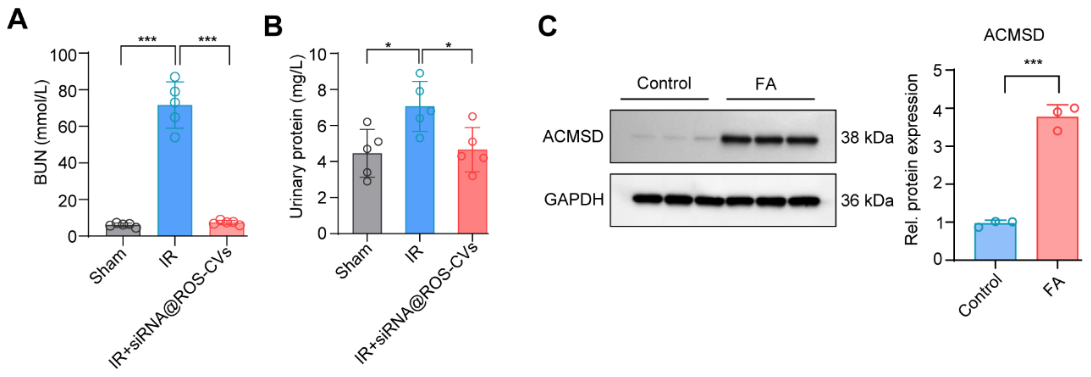


**Figure S22. The RNAi@ROS-CVs alleviates IR-induced kidney injury and the production of urine protein**. (**A**) The blood urea nitrogen (BUN) levels of AKI mice after different treatment ($n$ = 5). (**B**) The urine protein levels of AKI mice after different treatment ($n$ = 5). (**C**) The protein expression and statistical analysis of ACMSD in mice kidney after folic acid treatment ($n$ = 6). Data were presented as means ± sd. Tukey-corrected one-way ANOVA was used for statistical analysis in (**A**) (**B**). Two-tailed unpaired t test was used for statistical analysis in (**C**). ns: not significant, *$P < 0.05$, ***$P < 0.001$.

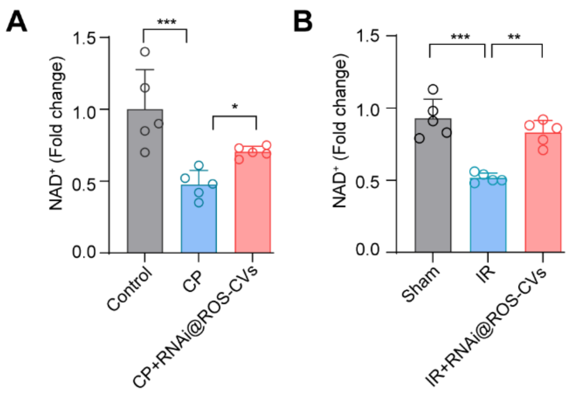


**Figure S23. The ACMSD inhibition rescued the renal NAD$^+$ levels *in vivo* when AKI occur**. (**A**) The renal NAD$^+$ levels in CP induced-AKI mice after different treatment ($n$ = 5). (**B**) The renal NAD$^+$ levels in IR induced-AKI mice after different treatment ($n$ = 5). Data were presented as means ± sd. Tukey-corrected one-way ANOVA was used for statistical analysis. ns: not significant, *$P$ < 0.05, **$P$ < 0.01, ***$P$ < 0.001.

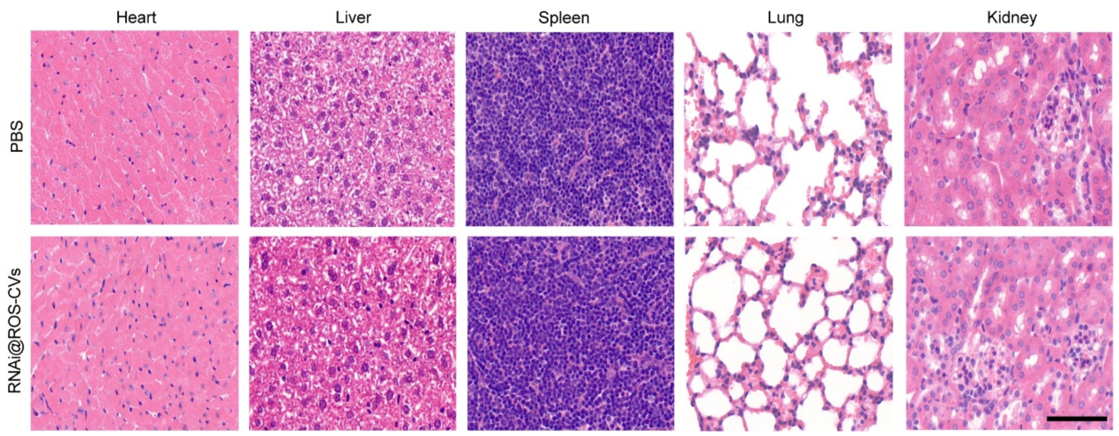


**Figure S24. Histopathology of major organs after RNAi@ROS-CVs administration**. Haematoxylin & eosin (H&E) staining of the major organs of mice after administration of RNAi@ROS-CVs for consecutive seven days. Scale bar: 50 μm.

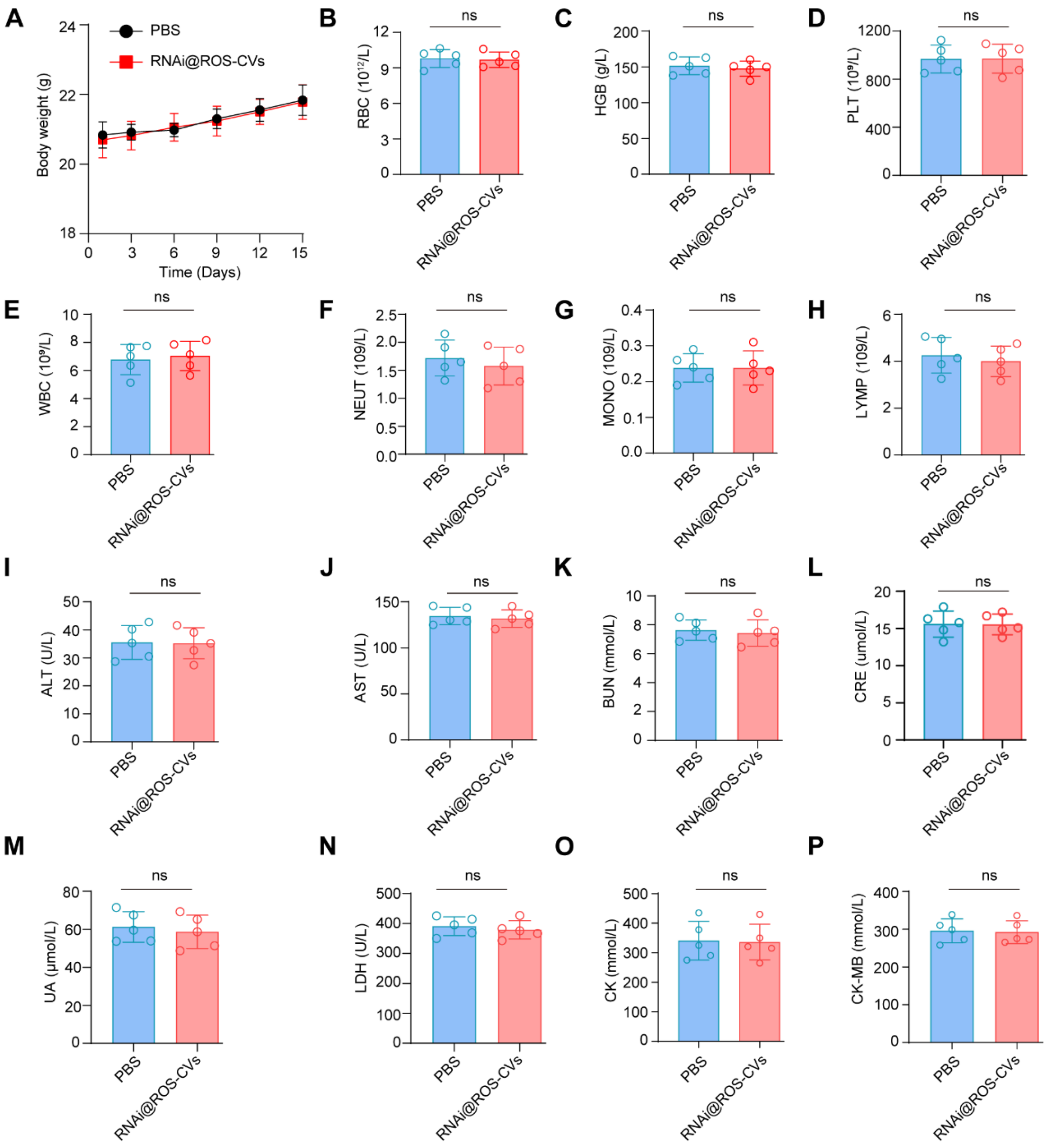

**Figure S25. Toxicity of RNAi@ROS-CVs administration**. (**A**) The body weight of C57BL/6 mice subject to RNAi@ROS-CVs or PBS treatment for consecutive seven days. (**B-H**) Blood routine (RBC, red blood cell; HGB, hemoglobin; PLT, platelet; WBC, white blood cell; NEUT, neutrophil; MONO, monocyte; LYMP, lymphocyte) of healthy C57BL/6 mice in different group. (**I-P**) Blood biochemistry analysis (ALT, alanine transferase; AST, aspartate transferase; BUN, blood urea nitrogen; CRE, creatinine; UA, uric acid; LDH, lactic dehydrogenase; CK, creatine kinase; CK-MB, creatine kinase-MB) of healthy C57BL/6 mice in different group. Data are presented as means ± sd. $n = 5$ per group. ns: not significant. Two-tailed unpaired t test was used for statistical analysis.

**Table S1. Sequences of the primers used for real-time PCR analysis.**

| Gene | Forward primer (5'→3') | Reverse primer (5'→3') |
|---|---|---|
| GAPDH_hQ | AGCGAGCATCCCCCAAAGTT | GGGCACGAAGGCTCATCATT |
| GAPDH_MQ | GGTGAAGGTCGGTGTGAACG | CTCGCTCCTGGAAGATGGTG |
| Ptgs2_hQ | ATGCTGACTATGGCTACAAAAGC | TCGGGCAATCATCAGGCAC |
| Ptgs2_MQ | TTCAACACACTCTATCACTGGC | AGAAGCGTTTGCGGTACTCAT |
| ACMSD_hQ | GGTGCGAGAGAATTGCTGG | TGCTGGCAAGGTCGTTGTTT |
| GPX4_hQ | AGGAGCCAGGGAGTAACGAA | AGCCGTTCTTGTCGATGAGG |
| GPX4_MQ | GATGGAGCCCATTCCTGAACC | CCCTGTACTTATCCAGGCAGA |
| NGAL_MQ | GCCCAGGACTCAACTCAGAA | GACCAGGATGGAGGTGACAT |
| KIM-1_MQ | GGAAGTAAAGGGGGTAGTGGG | AAGCAGAAGATGGGCATTGC |
